# Knowledge, compliance, and challenges in anti-malarial products usage: a systematic review of at-risk communities for zoonotic malaria

**DOI:** 10.1186/s12889-024-17792-8

**Published:** 2024-01-29

**Authors:** Nurul Athirah Naserrudin, Bipin Adhikari, Richard Culleton, Rozita Hod, Mohammad Saffree Jeffree, Kamruddin Ahmed, Mohd Rohaizat Hassan

**Affiliations:** 1 Institute for Health Systems Research, National Institutes of Health, Ministry of Health, Setia Alam, Shah alam, 40170 Malaysia; 2 Department of Public Health Medicine, Faculty of Medicine, Universiti Kebangsaan Malaysia, Kuala Lumpur, 56000 Malaysia; 3 Mahidol Oxford Tropical Medicine Research Unit, Faculty of Tropical Medicine, Mahidol University, Bangkok, Thailand; 4 Centre for Tropical Medicine and Global Health, Nuffield Department of Medicine, University of Oxford, Oxford, UK; 5 Division of Molecular Parasitology, Proteo-Science Center, Ehime University, Toon, Ehime, 791-0295 Japan; 6 Borneo Medical and Health Research Centre, Faculty of Medicine and Health Sciences, Universiti Malaysia, Sabah, 88400 Kota Kinabalu Malaysia; 7 Department of Public Health Medicine, Faculty of Medicine and Health Sciences, Universiti Malaysia Sabah, Kota Kinabalu, 88400 Malaysia

**Keywords:** Non-human simian malaria parasites, Zoonotic malaria, *Plasmodium knowlesi*, *Plasmodium inui*, *Plasmodium cynomologi*, Prevention, Malaria prevention, Knowledge, Community prevention, Southeast Asia, Western Pacific

## Abstract

**Background:**

Zoonotic malaria is a growing public health threat in the WHO Southeast Asia (SEA) and Western Pacific (WP) regions. Despite vector-control measures, the distribution of *Macaque fascicularis* and *M. nemestrina*, and *Anopheles mosquitoes* carrying non-human simian malaria parasites poses challenges to malaria elimination. The systematic review assesses the literature on knowledge and malaria-preventive practices in zoonotic malaria-affected areas across the WHO SEA and WP, aiming to identify challenges for malaria control.

**Methods:**

Peer-reviewed articles published in English, Malay and Indonesian between January 2010 and December 2022 were searched in OVID Medline, Scopus, Web of Science, and Google Scholar. Studies of any design—excluding reviews, conference proceedings, and reports from all WHO SEA and WP countries vulnerable to zoonotic malaria—were included. Backwards-reference screening and thematic analysis were conducted.

**Results:**

Among 4,174 initially searched articles, 22 peer-reviewed articles met the inclusion criteria. An additional seven articles were identified through backwards-reference screening, resulting in a total of 29 articles for this review. Half of these studies were conducted in Cambodia, Myanmar, Malaysia, and Thailand, mainly in forests and remote communities. The review highlighted inconsistencies in the operationalization of knowledge, and five major themes were identified related to knowledge: causation and transmission, symptoms, treatment, severity and complications, and malaria prevention. While participants generally had some understanding of malaria causation/transmission, minority and indigenous ethnic groups demonstrated limited knowledge and held misconceptions, such as attributing malaria to drinking dirty water. Preventive practices included traditional and non-traditional or modern methods—with a preference for traditional approaches to avoid mosquito bites. Challenges to malaria control included feasibility, cost, and access to healthcare services.

**Conclusion:**

This review provides insights into knowledge, local understandings, and preventive practices related to malaria in the WHO SEA and WP regions. The findings highlight the need for future research to explore the knowledge of at-risk communities regarding zoonotic malaria, their perceive threat of the disease and factors exposing them to zoonotic malaria. New strategies must be developed for zoonotic malaria programs tailored to local contexts, emphasizing the significance of community participation, health education, and socio-behavioural change initiatives. It is important to consider the interconnectedness of human health, environmental and non-human primates conservation. Socio-cultural nuances should also be carefully considered in the design and implementation of these programs to ensure their effect tailored to local contexts.

**Supplementary Information:**

The online version contains supplementary material available at 10.1186/s12889-024-17792-8.

## Background

Malaria elimination remains a global-health priority [1]. The efforts to eliminate human malaria such as *Plasmodium falciparum* malaria and *Plasmodium vivax* malaria has been successful and progressing positively in several regions in the world [2]. In 2021, China and El Salvador were certified malaria free due to zero indigenous cases reported for at least three consecutive years [2]. However, the emerging of zoonotic malaria in WHO South-East Asia (SEA) posing a significant challenge to malaria elimination efforts [1, 2]. Non-human simian malaria species such as *P. knowlesi, P. cynomologi, P. inui,, P. inui-*like, *P. coeatneyi*, and *P. simiovale* has been identified in indigenous communities living in forest-fringe in Malaysia [3]. Despite successful control of malaria in some countries, the SEA; i.e., Thailand, Myanmar, Indonesia, and Singapore [4] and Western Pacific region (WP); i.e., Malaysia, Vietnam, the Philippines, Cambodia, and Laos PDR continue to struggle with elimination measures due to natural reservoirs and malaria vectors [5–6]. As compare to human malaria, where the human to human transmission occurs through the bite female *Anopheles* species, the risk of acquiring zoonotic malaria is highest for people who live, do activities, or working at the forest fringe because of their close proximity with the monkey reservoir hosts and female mosquito vectors [3]. Long-tailed *Macaque fascicularis* and *M. nemestrina* monkeys, as well as *Anopheles* vectors, pose an ongoing non-human simian malaria risk to humans [4].

The emergence of *Plasmodium knowlesi* malaria, first reported in Sarawak, Malaysia, over a decade ago, remains a concern [7]. In Sabah, located in Malaysian Borneo the estimated incidence has reached 0.5 to ‍3.6 per 1,000 people [8], with a case fatality rate of 2.5 per 1,000 for women and 1.7 per 1,000 for men during 2010–‍2017 [9]. Shifting malaria epidemiology in these regions, with asymptomatic *P. knowlesi* malaria in humans, underscores the necessity of preventive measures to prevent outbreaks [10].

Vulnerable communities residing in forest or forest-fringe areas are vulnerable to zoonotic malaria [11]. To overcome these barriers, countries in the Asia Pacific have integrated community health care workers to facilitate government clinics in providing basic care and screening for febrile individuals [12]. Malaria subsectors have also been established in Malaysia to offer malaria care services in remote communities [13]. However, with the increasing detection of zoonotic malaria cases yearly, there is a need for new policy approaches in zoonotic malaria-prevention strategies [14–15].

In the WHO SEA and WP region, studies on zoonotic malaria have focused on socio-demographic factors [16], vector biology, behaviour, and distribution [17], and environmental factors [18], such as the distribution of *Macaque* monkeys [19]. However, limited understanding continues to pose a challenge to malaria prevention. According to the Knowledge, Attitude, and Practice (KAP) theory, knowledge can enhance attitudes and lead to positive behaviour formation, ultimately resulting in disease prevention [20]. Other studies have argued that the KAP theory insufficiently addresses behaviour and disease control because other factors, such as the environment and socioeconomic, political, and cultural factors, are equally important [21]. From a public-health perspective, disease prevention is essential for population health [22]. A holistic approach that considers all factors influencing population health is therefore needed. For malaria, this requires addressing the root causes of the disease by understanding the challenges of communities to improve health and overcome barriers to implementing culturally appropriate, scientifically sound, and financially feasible interventions [23]. It should be acknowledged that, certain population such as mobile and migrant populations (MMPs) in WHO SEA and WP regions are at-risk for both zoonotic and human malarias [24].

To address this critical knowledge gap, this study is an attempt to systematically compile literature on vulnerable populations in the WHO SEA and WP regions to better understand the local knowledge and understanding of malaria, preventive practises, and challenges to compliance with anti-malarial products. Given the significant threat of zoonotic malaria, to these populations, the information gathered from this review may guide future research and inform appropriate zoonotic malaria strategies. malaria.

## Methods

### Study concept and eligibility criteria

This systematic review followed the Population, Concept, and Content framework to ensure a comprehensive and systematic approach to article selection. Eligible articles were chosen based on the criteria listed in Table 1:

**Table 1:** The eligibility criteria

The study concept	The eligibility criteria
Population	The study population included individuals in the WHO WP and SEA regions exposed to zoonotic malaria
Concept	The study focused on the local understanding of malaria, such as knowledge, beliefs, and preventive practises
Content	This review considered original articles, such as case-control, observational, and cross-sectional studies, in addition to other epidemiological studies, which investigated the knowledge, beliefs, and preventive practises of communities exposed to zoonotic malaria. Due to the heterogeneity of terminology used in the malaria literature, both “perception” and “perspective” were utilized as search terms.

This systematic review was registered with PROSPERO under the registration number CRD42021251323, ensuring transparency and accountability in the review process.

### Search strategy

Five databases—PubMed, Ovid Medline, Scopus, EBSCOhost, and Google Scholar—were systematically searched for relevant articles using a range of medical subject headings terms, including “malaria,” “fever,” “febrile illness,” “mosquito-borne illness,” and “knowledge.” A backward reference search was also conducted to identify additional articles. The study focused on articles published between January 1, 2010 and December 31, 2022, since molecular screenings for non-human primate malaria parasites expanded during this period following the detection of *P. knowlesi* malaria in Sarawak, Malaysia, in 2004 [7]. Non-peer-reviewed articles, conference proceedings, animal studies, study protocols, literature reviews, and reports from governmental and non-governmental organizations were excluded from this review because they were difficult to assess for quality using the checklist criteria. Studies that addressed preventive practices, but not the knowledge and beliefs of communities in the SEA and WP regions were also excluded. To capture any missing articles, a manual search was performed using the Google search engine [25]; only the first 200 results were screened for relevance to this review.

### Screening strategy: study selection, data extraction, and result synthesis

The study selection and data-extraction process were rigorously and systematically conducted and involved three stages.

In the first stage, the titles and abstracts of the searched articles were screened by N. A. N., R. H., and M. R. H.

In the second stage, the same reviewers screened the full text of the articles and selected those that met the eligibility criteria. Any disagreements between the reviewers were resolved through consensus by K. A. and M. S. J. To minimize errors and bias, the reviewers extracted and organized data from the selected studies into a table, which included the first author’s name, the year of publication, the area of study, the methodology used, the study participants, knowledge and beliefs, and preventive practises. After resolving disagreements, the extracted data were compared and merged into a single database, and all duplicates were manually removed.

In the third stage, the authors evaluated the eligibility of the selected studies based on the distribution of *M. fascicularis* and *M. nemestrina* monkeys and *Anopheles* species that harbour *P. knowlesi*, *P. cynomologi*, *P. inui*, and other non-human primate malaria parasites, all of which increase the risk of zoonotic malaria [26]. Areas with a low risk of zoonotic were excluded.

Finally, the authors reviewed the references of all remaining studies to identify any additional eligible studies, and K. A. and M. S. J. resolved any disagreements between the reviewers.

### Quality appraisal

The quality of the eligible studies was independently assessed by three co-authors: N. A. N., R. H., and M. R. H., using the Joanna Briggs Institute Critical Appraisal Tools to ensure the reliability and validity of the study findings. The quality assessment results are presented in Additional Table 1, and provide transparency and clarity on the quality of the included studies. The outcome of the appraisal did not affect study inclusion.

### Thematic analysis

The primary author (N. A. N.) identified and clustered similar meanings into codes and themes [27]. The generated themes were reviewed and defined with M. R. H. and R. H to increase the richness of the findings.

## Results

**Fig. 1:**
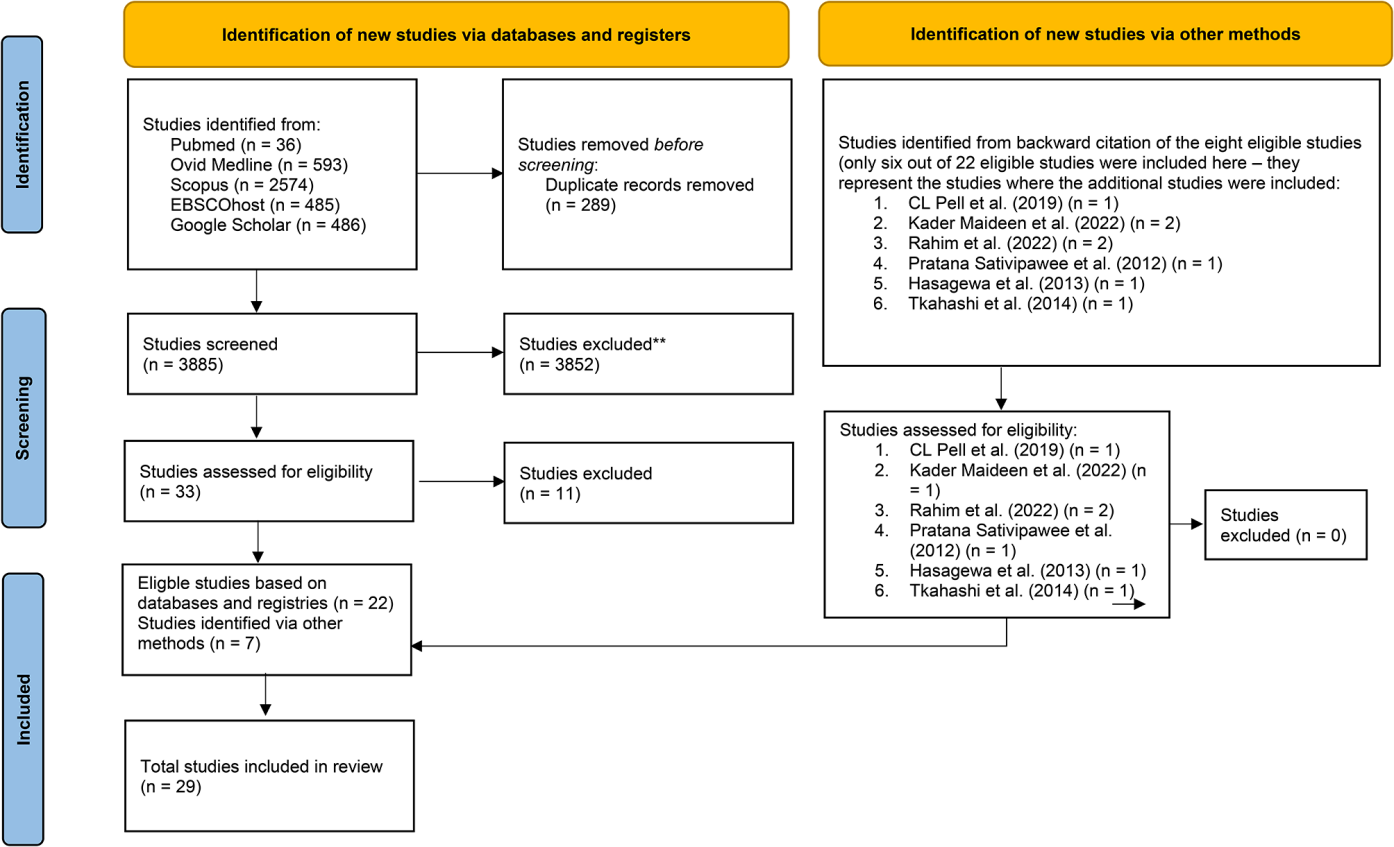
The PRISMA 2020 flowchart of articles in the review. *Note:* Page MJ, McKenzie JE, Bossuyt PM, Boutron I, Hoffmann TC, Mulrow CD, et al. The PRISMA 2020 statement: an updated guideline for reporting systematic reviews. BMJ 2021;372:n71. doi: 10.1136/bmj.n71

The initial search of 4,174 articles resulted in the inclusion of 22 articles after duplicates were removed. The included articles were obtained from five databases—PubMed, Ovid Medline, Scopus, EBSCOhost, and Google Scholar—and seven additional studies were selected after a backwards-citation search of the references for the eligible studies. In total, 27 articles were included in this review. (Fig. 1, Additional Table 1. The eligible articles)

Several studies were excluded for various reasons, including a lack of information on malaria knowledge, duplications of eligible studies, participants who were immigrants rather than residents or workers in the study area, community-engagement studies with less of a focus on community knowledge, and because the study area had a lower risk of zoonotic malaria.

### Variations in study methodology

There were ten quantitative studies, three qualitative studies, nine mixed methods (MM) studies, and other study designs (e.g. multimethod and quasi-experimental design). The methods, study population and findings are described in Additional Table 1. The study designs, methods, study populations, and contexts were heterogeneous, so a meta-analysis was inappropriate [28].

The studies were conducted in eight countries in the WHO WP and SEA regions, including Cambodia [29–34], Malaysia [35–37], Lao PDR [38–39], the Philippines [40–41], Vietnam [42] Myanmar [43–47], Indonesia [48–51], Thailand [48–50, 52–54]]; and multiple sites in Myanmar, Laos PDR, Cambodia, and Vietnam [55]. All the studies were conducted in rural areas, often near or within forests, and most areas were remote, with limited healthcare services.

While all studies were conducted in areas where communities were vulnerable to zoonotic malaria, only three studies investigated the presence of monkey sightings using surveys [36–37] and interviews [36, 50] According to forest workers in Aceh, Indonesia, there were frequent sightings of monkeys at their workplaces [50]. The indigenous respondents in Peninsular Malaysia were generally aware of malaria, but there were no articles mentioned whether the communities were aware of ‘monkey malaria’. Consequently, there is no evidence demonstrating whether these communities perceive ‘monkey malaria’ as a health threat, or view the forest as a risk area for zoonotic malaria infection.

More than 92% of the studies had adult participants, and only two studies recruited children [37, 41]. The age range of the study participants varied, with the youngest being six years old [37]. Most participants were adults 18 years of age or older, although the mean age varied considerably across studies, and not all studies included data on the study participants’ average ages.

While the study populations were generally well-balanced in terms of gender, some focused exclusively on females [33], had a higher proportion of female participants [36–37, 54, 56], or were comprised of more males than females [51].

Some studies focused on specific groups, including rubber tappers [43, 52], mobile and migrant populations (MMPs) [29, 31], forest-goers [32, 34, 54], community health workers (CHWs) [40, 57], village malaria workers (VMWs) [30–31, 57], village health volunteers (VHVs) [44–45], and military personnel ( [38]. Several studies recruited indigenous communities such as Orang Asli aboriginals [36–37] and ethnic minorities such as the Raglai [42] and Khmer ethnic as study participants [33].

These communities were at high risk of malaria due to various sociocultural activities, work habits, and places of work or residence. These include outdoor activities like farming ‌and working on oil palm, rubber plantations and coconut plantations and other forest-related work (e.g. rice cultivation, wood cutting, hunting, foraging, fishing, small-scale subsistence slash-and-burn agriculture and mining. These activities often required workers to remain in the forest, thereby increasing their exposure to malaria. For instance, children of mothers from minority ethnic groups in Cambodia who stayed in the farm huts have nearly four times higher odds of experiencing fever in the last three months compared to those who did not stay at the farm hut (aOR 3.681; 95% CI 1.943–‍6.972) [33]. Social activity, such as gathering outside the home, was common among the indigenous communities in Peninsular Malaysia residing along the central spine forest range area [36].

The sample sizes in this review varied considerably and were categorized based on previous studies [58]. Of the 29 studies included in our analysis, four qualitative-design studies had a small sample size of fewer than 100 participants [34, 49–51], ‌‌ 17 had a medium sample size of more than 100 but less than 600 participants [30–31, 33, 35–40, 43–45, 48, 51, 53–54], three had a medium-large sample size of more than 600 but fewer than 900 participants [32, 53, 57],three had a large sample size ranging from 900–‍1,999 participants [29, 55, 59],and three had a very large sample size of more than 2,000 participants [42, 46–47]. The study in our analysis with the largest sample size was conducted in a malaria-endemic region in Myanmar and included 6,597 participants [46].

### Knowledge and local understanding of malaria

This systematic review identified the following five major themes from the eligible articles: knowledge of malaria causation and transmission, including mosquito species, vector biting time, and the parasite species responsible for malaria; knowledge of malaria symptoms; knowledge of malaria diagnosis and treatment, including anti-malarial drugs and drug resistance; knowledge of malaria complications; and knowledge about malaria prevention (Table 2).

**Table 2:** The themes and their definitions

Themes related to malaria knowledge	Definition
Malaria transmission and causation	This theme includes knowledge about malaria transmissions, such as mosquito species, the time of day they bite, and the parasites involved in transmission.
Malaria symptoms	This theme includes knowledge of malaria symptoms and how those symptoms present in infected individuals.
Malaria diagnosis and treatment	This theme includes knowledge about malaria diagnosis and treatment, such as anti-malarial drugs and drug resistance.
Malaria complications	This theme includes knowledge about the potential complications that can arise from malaria infection, such as severe anemia, cerebral malaria, and respiratory distress.
Malaria prevention	This theme includes knowledge about methods for preventing malaria infection, such as using ITNs, mosquito repellents, and prophylactic medications.

Each study investigated different dimensions of malaria knowledge among participating populations. A study on Palawan Island in the Philippines, for example, explored knowledge of malaria transmission, vector species, and vector biting time [40]. Other studies covered information on various topics, such as knowledge of asymptomatic malaria [39, 55], malaria control among village workers [44], drug resistance [29, 38], malaria risk in villages, and elimination of malaria [35, 39, 59].

The understanding of malaria varied depending on the location of the communities. Some recognized malaria as a local health concern and a common disease [34, 51, 55]. They believed most people, such as community members and families, had been diagnosed with malaria [32]. Studies conducted between 2018 and 2022 revealed misconceptions about malaria transmission. Nearly 90% of respondents in Myanmar had previous experience with malaria and were familiar with the word “malaria” [51] and 72% of respondents in the Alue Bilie subdistrict of Aceh, Indonesia, understood the word “malaria” [49]. In Western Cambodia, most participants knew “malaria” (98.6% in Pailin and 99.6% in Veal Veang) [29]. However, in Vietnam, half of the participants were uncertain about the word “malaria,” instead referring to it as *sot ret* (“hot fever”) in Vietnamese or *lah sakih* in Ra-glai [42].

Perceived susceptibility to malaria varied among different populations. Military personnel in Laos PDR recognized malaria as the second most common health issue after dengue fever [39]. Concerns about becoming infected were linked to economic consequences [45, 55]. Several studies confirmed that participants understood they could be at risk when they visited the forest, mountains, and streams [34, 55]. Some participants believed malaria was unavoidable due to the abundant mosquitoes in the forest [34], but preventable and treatable [35, 46]. Study participants viewed malaria as a burden and suggested increasing awareness and knowledge of malaria through various programs [52]. Some participants on Palawan Island in the Philippines demonstrated a biomedical understanding of malaria, which can be attributed to the numerous malaria control-and-elimination activities in the region [41]. Finally, malaria-affected volunteers with low-to-moderate knowledge scores of the disease faced a 2.4-fold greater risk of contracting the disease than those with higher knowledge scores (*aOR* = 2.4; 95% CI: 1.1–‍5.0, *p* < 0.05) [52].

### Malaria knowledge factors

This review identified various factors that influence knowledge of malaria. In Kelantan, Malaysia, age, education, and ethnicity were significant factors (*p* < 0.001), with the 19–‍40 age group demonstrating correct knowledge of malaria, which suggests age is a critical factor in malaria knowledge [37]. Education level also contributed to the malaria knowledge of the participants in Pahang (*x*^2^ = 4.244, *p* = 0.039), with better-educated individuals having a better understanding of malaria and the symptoms (*x*^2^ = 24.037, *p* < 0.001 and *x*^2^ = 4.416, *p* = 0.036, respectively) [35].

In Myanmar, poor knowledge about malaria was associated with poor treatment-seeking behaviour (2.9, 95% CI = 1.6–‍6.2, *p* < 0.001), with those above 60 years of age (9.0, 95% CI = 6.0–‍17.2, *p* = 0.01), females (1.5, 95% CI = 1.0–‍1.9, *p* = 0.03), and illiterate individuals demonstrating poor health-seeking treatment behaviour (3.5, 95% CI = 2.0–‍7.8, *p* = 0.0018) [46]. Good knowledge of malaria among the Orang Asli in Peninsular Malaysia was positively correlated with attitude (*r* = 0.346, *p* < 0.001) and practice in avoiding malaria (*r* = 0.236, *p* < 0.001). However, no correlation was detected between attitude and practice, which suggests better malaria knowledge does not necessarily translate into better attitudes and/or practises toward malaria prevention [36].

In the Philippines, knowledge about malaria transmission was the most significant aspect of knowledge of malaria [40]. However, knowledge of malaria was negatively correlated with attitudes and practises associated with the disease in some cases [56], This highlights the importance of understanding each community’s unique context when considering barriers to improving attitudes and practises toward malaria prevention.

### Knowledge of malaria causation and transmission

#### Natural causation of malaria and knowledge of its transmission

Multiple studies showed the majority of study participants understood that mosquito bites were the primary cause of malaria.‌ In Kelantan and Pahang, Malaysia for example, 51.9% and 70.7% of the participants, respectively, had a clear understanding of the link between mosquitoes and malaria [37]. Besides these two states, participants in Laos PDR also recognized the risk of contracting the disease when working in the forest [38].

However, some studies have found that less than half of the participants understood how malaria could be transmitted. For instance, only 47.7% of military personnel in southern Laos PDR knew malaria was transmitted through *Anopheles* mosquito bites, but many confused it with dengue fever [38]. While villagers in Vietnam were generally aware of the role of mosquitoes that bite at night in malaria transmission, they had a limited understanding of the timing of mosquito bites due to the higher visibility of mosquitoes in the evening and early morning [42]. In addition, aboriginal and rural communities in Peninsular Malaysia had limited knowledge about the causative agent of malaria and how mosquitoes acquire the parasite [35]. Similarly, a low percentage of mothers in Cambodia with children under two years of age could correctly identify mosquito breeding places (29.2%); mosquito species (23.9%); the life cycle of mosquitoes (3.4%); and malaria transmission (40.6%) [33].

#### Misconception on the causation of malaria and knowledge of its transmission

Misconceptions about malaria transmission were prevalent among the ethnic minority groups and villagers included in the studies. Some respondents associated malaria transmission with poor hygiene and dirty surroundings [29, 31, 37, 42, 46], consumption of contaminated food and water [29, 31–32, 46] drinking streams or un-boiled water [55], and eating bananas [46]. Other perceived contributing factors were forests, excessive sun exposure and hard work, stagnant water, sharing shelter and sleeping together, and sleeping without bed nets. Some believed coughing or sneezing could spread malaria or be transmitted by insects such as flies [42, 55]. Certain beliefs related to biology included the belief that having the same blood type [46], a lack of red blood cells [42], and poor nutrition could increase the risk of malaria [37]. Weather and working in the sun were also believed to contribute to malaria transmission. Finally, some respondents believed in supernatural causation, such as malaria being caused by spirits [29].

Despite the implementation of malaria programs, studies revealed the presence of incorrect local beliefs about malaria. Malaria was associated with internal bodily states that were too hot, cold, or damp, attributed to factors such as the sun, hunger and living conditions [41]. Additionally, participants shared the potential sources of malaria, such as consuming dirty water or food and breathing in dirty air. Saliva was seen as a possible source of malaria [41]. Swimming in water contaminated with mosquito larvae was also mentioned as a possible means of contracting the disease. Furthermore, participants recognized external agents, such as dirt introduced through contaminated water and food or direct contact with soiled items, as contributing factors to malaria transmission [41].

The community in Laos PDR understood malaria to be a disease caused by lifestyle and place rather than parasites. More than half (55.2%) of the Raglai ethnicity in Vietnam did not know malaria causes [42].

Inaccurate beliefs and inadequate knowledge about malaria pose significant challenges to effective prevention efforts, as individuals with inaccurate beliefs about malaria are at a higher risk of contracting the disease [50, 52].

### Knowledge of malaria symptoms

Most studies found community participants could identify common malaria symptoms, such as fever [29–31, 38, 41, 50], headache [31, 38, 50], and body pains [29–31, 38, 41, 50]. Some groups, however, such as Khmer women [33] and the Orang Asli in Malaysia, had lower awareness; for example, only 40.6% of the Orang Asli participants associated fever with malaria [37]. Interestingly, in Cambodia, people in villages with village malaria workers had less knowledge of malaria symptoms than in villages with healthcare [30]. Furthermore, indigenous Orang Asli in Lipis, Malaysia, with a history of malaria, knew more about malaria symptoms than those without a history of malaria (*x*^2^ = 6.810, *p* = 0.009) [35]. Recognizing malaria symptoms was emphasized as the first step to promoting community treatment-seeking behaviour [30].

### Knowledge of malaria diagnosis and treatment

The most common diagnosis known to study participants was blood tests, either through microscopy screening [31, 33, 41, 52, 57, 59] or a rapid-diagnostic test (RDT) [41, 59]. In Thailand and among indigenous communities residing in the central spine forest of Peninsular Malaysia, the importance of promptly performing a blood test if someone develops a fever, headache, and myalgia was well understood [36]. However, in remote areas of the northern Shan State in Myanmar, access to malaria screening using RDT and healthcare services was limited, resulting in healthcare providers offering treatment without blood tests [59].

Despite residing in rural areas of Myanmar, a high proportion (90%) of surveyed communities believed malaria could be treated, and 85% believed modern medicine could effectively treat the illness [46]. Nearly 80% of the military personnel in Cambodia understood that malaria is preventable and curable [38]. VMWs understood that patients could relapse if they failed to complete the malaria treatment regimens, and 90% of the villagers in that area were prescribed modern medicine at healthcare facilities [31]. Among the MMPs in Western Cambodia, 40.4% of the population with fevers sought medical assistance from VMWs, 27.9% from private clinics, and 20.2% from private clinics [29]. Of those receiving treatment from the VMWs, 75% sought help within one day of the onset of symptoms [29]. However, those who delayed medical assistance for two or more days after the illness onset faced an increased risk of developing complications [29]. This can be partly explained by the fact that VMWs training is focused on diagnosis and treatment rather than prevention and vector control [29]. A similar situation was described in Myanmar, where those with poor knowledge of malaria also delayed seeking diagnosis and medical intervention [45].

Most patients with malaria-like symptoms were treated with anti-malarial drugs [35, 54]. More than half (60.0%) of the participants in Thailand sought treatment when they developed symptoms [54]. While only a small proportion of participants in Cambodia purchased medication from a local shop (3.2%), nearly half could not recall the name of the anti-malarial drug [30]. Among those who could remember which anti-malarial drug they had taken, A + M was the primary anti-malarial drug, followed by Malarine [30]. Among the school children who participated in photovoice research on Palawan Island in the Philippines, one participant shared knowledge concerning Coartem, a primary malaria medication, and emphasized the importance of consulting medical personnel before consuming anti-malarial drugs [41].

In certain areas—such as Indonesia; indigenous people in Kelantan, Malaysia; and migrants and villagers living in remote areas of northern Shan State in Myanmar—still practised home and self-medication before considering medical care because they preferred traditional medicine [37, 48, 50, 59]. In Aceh, Indonesia, and throughout Cambodia, modern medicine was often viewed as an alternative option when traditional healers could not cure an illness [48, 50]. It was also determined that some individuals purchased a commercial brand of chloroquine from local pharmacies and consumed it without being tested for malaria [50]. Notably, individuals in Cambodia who did not comply with anti-malarial regimens were more likely to experience malaria relapse [31]. There was no further detail on the non-trade names of the drugs given in the studies.

Several factors influenced health-seeking behaviour, such as knowledge [35], past experiences with febrile illnesses and knowledge of malaria symptoms [29], proximity and accessibility to healthcare facilities [32, 50], the geography of a given area—specifically remote and hilly locations with poor access to health facilities [38, 45, 50, 59] —and the cost of transportation [50]. Others linked malaria health-seeking treatment with the perceived quality of healthcare [32, 59]; the efficacy of care [32]; a lack of medical personnel and inadequate health facilities [38, 59], negative experiences with healthcare providers [32, 50], and the absence of local health insurance [50]. Working adults also faced challenges seeking treatment due to their inability to do so while working [45]. The availability of healthcare services dependent on the opening time of area health facilities underscores the importance of VMWs as a source of care, followed by public health facilities [32–33].

Trust, good prior relationships, and the friendliness of healthcare service providers were significant factors influencing Myanmar communities to seek healthcare [59], in addition to the availability and cost of services and trust in healthcare services in Cambodia [32]. The language barrier was also identified as a significant factor in communities’ preferences to seek healthcare, where a study in Myanmar reported that difficulty in communicating with healthcare workers who did not understand local languages is a challenge [59]. Pregnant women were identified as a vulnerable population in Cambodia, where half of the respondents from VMW villages recognized the danger of malaria to their health [30]. It is concerning that nearly half (40%) of pregnant ethnic minority women in Cambodia did not seek treatment for fever, which highlights the need for increased awareness of health care services in these communities [33].

### Knowledge of malaria complications and severity

Malaria is perceived as a significant threat, particularly among military personnel in Laos PDR and MMPs in Cambodia [29, 38]. In Laos PDR, 93.8% of military personnel feared contracting malaria due to its potential severity and lethality [38]. Similarly, in Cambodia, 79% of MMPs perceived malaria as a significant threat, and 40% of forest-goers believed the disease to be harmful and difficult to cure [32]. The impact of malaria on livelihoods was also noted, as it could prevent individuals from working and earning money [32].

Insufficient knowledge of malaria was also associated with negative attitudes, poor practices, and a history of malaria relapse [48]. For instance, participants in Laos PDR were not aware that a seemingly healthy person could have malaria parasites in their blood, as only 14.2% of respondents understood the concept of asymptomatic malaria parasite carriage [38]. More than half (51.9%) of the respondents disagreed that a healthy person could carry the malaria parasite in their blood, and one-third (33.8%) were unsure about such a condition [39]. While half of the trial participants and the healthcare workers in Cambodia believed malaria was possible without developing symptoms, forest-goers did not accept this possibility [34].

### Knowledge of malaria prevention

The participants in these studies demonstrated a comprehensive understanding of malaria-prevention methods. In Cambodia, the use of bed nets and the importance of avoiding mosquito bites were identified with almost 100% accuracy, while the benefits of mosquito coils and protective clothing were also recognized [29, 57] ‍. Mass drug administration was also mentioned as a potential solution for malaria elimination [55].

In Palawan, the Philippines, study participants understood the importance of sleeping under bed nets; 77.4% returned home before dawn, and 69.9% wore protective clothing, long-sleeved shirts, and pants to avoid mosquito bites, while only 15% always carried hammock nets when going into the forest [40]. Meanwhile, the rural community in Pahang, Malaysia, had better knowledge of insecticide use for mosquito breeding site elimination than the aboriginal population (*x*^2^ = 23.136, *p* < 0.001) [35]. In Myanmar, migrant rubber tappers were able to correctly describe mosquito bites or mosquitoes as the cause of malaria. They outlined various methods to prevent mosquitoes, such as using mosquito coils, wearing long clothing, and cleaning the surroundings [43]. VMWs and mobile-and-migrant workers (MMWs) working toward eliminating malaria in Cambodia voiced concerns about demotivating issues in malaria prevention, including limited access to resources such as petrol, transportation, satchels, and bags, and insufficient tools to provide information and education to deliver key malaria messages to communities [31].

### Practises for preventing malaria and challenges to compliance

This review categorized malaria-prevention measures into traditional and non-traditional practices. The use of ITNs and LLINs was cited in several studies. Other methods such as mosquito coils [43], repellents, protective clothing, the elimination of breeding areas, and taking drug prophylaxis were also reported [34, 38, 46]. Traditional practices to avoid mosquitoes included building fires [32], wearing long-sleeved clothes, balaclavas, and gloves [34, 40] and sleeping under mosquito nets in the forest [34]. Studies also documented drug prophylaxis and the use of both chemical and non-chemical methods to combat malaria [34, 43, 46].

Several factors influenced malaria prevention practises. In Pahang, Malaysia, for instance, rural inhabitants and aboriginals’ practise were influenced by level of education, age, and race (*x*^2^ = 4.634, *p* = 0.031; *x*^2^ = 5.483, *p* = 0.019; and *x*^2^ = 7.965, *p* = 0.019, respectively); as well as access to health information and the ability to communicate with health staff [35]. In Palawan Island, Philippines, satisfaction with microscopists was a significant factor in malaria prevention among participants engaged in awareness-raising activities [40]. Conversely, no significant associations were detected between knowledge of vector species and the most active time of vectors with malaria prevention (ANOVA = 0.142 and 0.371, respectively) [40].

In the Mandailing Natal district of Indonesia, certain factors were associated with a higher risk of contracting malaria. These included being male, having a low level of education, working as a farmer, earning a low income, and living in poorly ventilated houses (*p* values = 0.02; 0.04; 0.04; 0.04; and 0.04, respectively). Poor knowledge and negative attitudes also contributed to malaria recurrence or relapse [48].

Certain factors in Cambodia, Palawan Island, and Greater Mekong Subregion influenced individually adapted malaria prevention practices. These factors included recognizing malaria symptoms, having access to media campaigns *via* billboards, radio, and television, receiving advice from family members, village health volunteers and health staff, and the affordability of malaria preventive products [29, 48, 52].

Malaria prevention practises are driven or hindered by various factors. Despite good knowledge as described above, malaria-prevention behaviour remained suboptimal in many cases, with personal experiences, economic consequences, and community beliefs and practises influencing adherence to prevention measures and other underlying factors as described in Table 3.

**Table 3:** Drivers and barriers to malaria prevention practises

Malaria prevention practises		References
**Drivers**		
Intrapersonal and interpersonal factors: individual and social drivers that influence malaria prevention behaviours and practises		
	Having a strong desire to protect oneself and/or one’s family from mosquito bites	[34, 36, 43, 46, 51]
	Having knowledge on malaria and being aware of the disease	[29–31, 35, 38, 40, 43, 48, 57]
	Having a prior history of being diagnosed with malaria	[33–34]
Socioeconomic influence: the presence of social and economic aspects of individual and communities that impact malaria prevention. For example, Absence from work when diagnosed with malaria (e.g. financial implications and productivity losses)		[32, 45, 55]
Social support: Factors of influences that facilitate and promote effective malaria prevention within the community		
	Health education and communication: having access to information on malaria and malaria campaigns in the community	[29, 48, 51–52]
	Perceiving mosquito control products (e.g. bed nets and LLIN/LLIHN) as a community’s social norm	[32, 38, 45, 55]
	Availability and accessibility to malaria control products	[29, 51–52]
**Barriers**		
Intrapersonal and interpersonal factors: The individual level and social-level factors that influence challenges in malaria prevention		
	Fatigue after work	[50]
	Low perceived risk of malaria and limited awareness of its severity	[42, 48, 52]
	Cultural beliefs and misconceptions about malaria	[29, 31–34, 37–39, 41, 45–46, 48, 50, 52, 55]
Barriers related to design of bed nets/LLINs/LLIHNs: the challenges or obstacles arising from these mosquito control products’ specific characteristics or features.		
	Discomfort and usability when sleeping under bed nets	[50–51]
	Difficulty in breathing	[50]
	Discomfort due to heat retention under the bed net	[32, 50]
	Discomfort with the smell of bed net	[32, 51]
	Irritation or discomfort in the eyes when using bed nets	[50]
	Limited mobility or restricted movement while using bed nets	[43, 51]
	Experiencing a sense of claustrophobia when using bed nets	[51]
	The bed nets are worn out	[42]
	Not user-friendly to bring and use the bed net in forest or work area (e.g. logging; mining; gathering rattan; and forest patrol)	[32, 48–49]
Personal practices: Highlighting the individual actions, behaviours, and choices that individuals adopt, which can either increase or decrease their vulnerability to malaria		
	Preference for wearing regular clothing at home or work rather than using bed nets while sitting or sleeping	[43]
	Preference for alternative mosquito control methods, such as using fish, mosquito coils, or protective clothing	[32, 43, 51]
	Preference for traditional practices or methods	[37, 48–49, 59]
Healthcare-related factors: Refer to various aspects of the healthcare system that can influence malaria prevention efforts		
	Limited access to healthcare services, including malaria intervention and health messages	[32–33, 38, 45, 50, 59]
	Healthcare workers-related factors	[29, 32, 50, 59]
	Lack of trust of healthcare providers	[32]
	Lack of ownership or limited purchasing power (e.g., the high cost of mosquito control products)	[42]
	Absence of local health insurance to cover for healthcare cost	[50]
Insufficient social support: lack of support and assistance from the government, non-governmental sectors or communities in promoting and implementing effective malaria prevention measures		[45, 55]
Influence of weather conditions: refers to people’s willingness or ability to use mosquito control products for malaria prevention		
	Concerns about high temperatures and humidity that may lead to discomfort when using bed nets	[42–43, 51]
Fear: Presence of emotional response that individual experiences concerning malaria prevention		
	Fear of their bed catching fire while sleeping under the bed net, mainly when using fire for warmth in cold and humid environments.	[42]
Living experience: Various conditions and circumstances of people’s lives that can impact their ability to adopt and sustain preventive measures against malaria		
	No instances of malaria infection within households.	[52]
	Perceived the usage of mosquito control products as unnecessary due to the low incidence of the disease	[37]
	Socioeconomic conditions (e.g. Impoverished households, families lacking blankets resort to using bed nets as covers while sleeping, and bed nets are frequently damaged due to poor living conditions.	[42]
Social norm: non-verbal rules that are widely accepted, understood, and followed within the community		
	The infrequent usage of mosquito control products in the area or the lack of social acceptance towards malaria preventive measures	[32]

### Traditional practises

Traditional practices included removing stagnant water and emptying water vessels [35–36, 43], closing windows and doors during peak mosquito biting hours [36], cutting bushes around houses to reduce mosquito breeding areas [36], cleaning outdoor surroundings, including streams, and water sources [32, 36, 38, 49], building a fire and creating smoke [38, 49], using medicinal plants [35], believing in witchcraft and sorcery to treat febrile diseases [35], and practising personal hygiene [32]. Forest-goers in Cambodia built fires at night and used LLINs and long-lasting insecticide hammock nets (LLIHNs) because they believed fire repels mosquitoes [32]. Study participants in Sumba, Indonesia, use various approaches with papaya leaves to prevent malaria, such as putting them under the bed and consuming papaya leaves as a traditional method to treat malaria [51]. Participants preferred to drink boiled neem, betel and papaya leaves, then consume medicines from the hospital. Certain commercialized products such as *Nona Mas, Autan, Soffell*, and *Telon* oils were rubbed into the skin to avoid mosquitoes [51].

### Non-traditional and modern practises

Non-traditional and modern malaria-prevention practices include ITNs, LLINs, protective clothing, mosquito coils, insect/mosquito repellents, hammocks, and prophylactic drugs. A high percentage of participants in Thailand reported high compliance and use of bed nets and LLINs [52, 54]. Similarly, a high percentage of bed nets and LLINs (89.2%) was noted among aboriginals in Kelantan, Malaysia, with 95.2% reporting high compliance and usage. In contrast, compliance with using bed nets, protective clothing, mosquito coils and insect repellents was low among these populations, with only 50.0%, 14.1%, 4.9%, and 2.7% reported usage, respectively [37]. The rural community in Pahang, Malaysia, practised the use of insecticides and eliminated breeding sites to a significant extent compared to the rural aboriginals in the same area (*x*^2^ = 23.136, *p* < 0.001) [35].

Amongst military personnel in Laos PDR, high percentage rates were reported for various preventive measures, including mosquito repellent (91.3%), bed nets (90.0%), mosquito coils (87.8%), and prophylactic drugs that were distributed in the camps (73.6%) [38]. The use of ITNs and protective clothing was common in Alu Billie, Indonesia [49], but bed nets were irregularly used in Vietnam and not all family members slept under them [42]. The three methods preferred by MMPs in Cambodia to prevent malaria were LLINs/LLIHNs (70%), mosquito repellents (55% spray, 46% lotion), and mosquito coils (46%) [32]. Most mothers of children under two years of age slept under bed nets at home (95.8%) and wore long-sleeved clothes (83.8%) to prevent malaria. Mothers who took malaria-prevention actions were associated with a reduced risk of fever in their children (AOR 0.292; 95% CI: 0.136–‍0.650) [33].

A study in Thailand identified physical, chemical, electrical, and fumigation methods to prevent malaria, but the effectiveness of each could not be determined [51]. While the heterogeneity of the usage of different items was noted, studies using quantitative and qualitative methods have demonstrated a better understanding of communities’ perspectives on malaria [32]. Most aboriginals in Perak, Malaysia, did not add larvicides to stagnant water (82.0%) or use mosquito repellents (71.5%), indicating a potential prevention-effort gap [36]. While mosquito repellent was a preferred method to prevent malaria among forest-goers in Cambodia, they had limited knowledge of repellents, including where to obtain them, reflected by only 9% reporting regularly using the repellents [32]. Adaptations made by rubber tappers in Myanmar, such as tucking mosquito coils into their headband or waistband and wearing traditional sarongs (*htamein*) may provide insights into ways to improve malaria-prevention strategies in other settings [43].

## Discussion

We reviewed the knowledge and local understanding of malaria among communities in the WHO SEA and WP regions exposed to zoonotic malaria. We identified knowledge gaps in malaria understanding among at-risk communities and malaria-prevention challenges, highlighting areas where further research is needed. We also identified themes relating to knowledge concerning malaria transmission, symptoms, diagnosis, treatment, complications and disease severity. The absence of a standard definition for measuring and reporting knowledge of malaria leads to inconsistencies, making it challenging to assess the quality and reliability of the evidence. Despite this limitation, the utilization of thematic analysis enabled the identification of meanings and patterns across studies [27], concerning knowledge (Table 2) and the identification of drivers and barriers to preventive practises (Table 3). Moreover, the absence of a standard definition may reflect broader conceptual and sociocultural differences in how malaria is understood and perceived in different populations. This highlights the need for further research and discussion to develop more socioculturally sensitive malaria interventions and public health strategies in these regions.

Our findings emphasize the critical need to comprehend the diverse knowledge and perceptions surrounding malaria within different populations. Understanding the challenges associated with preventionis crucial for designing targeted interventions. A one-size-fits-all, top-down approach may be irrelevant locally, as demonstrated by various barriers to malaria prevention practises (Table 3). Therefore, initiatives aimed at understanding community’s knowledge and preventive practices must carefully account for these contextual factors. Involving communities in the identification of health can be instrumental in developing locally-relevant interventions suitable for local conditions. This collaborative and inclusive approach can facilitate the creation of sustainable prevention strategies. Engaging communities in this process fosters a sense of ownership, potentially enhancing the effectiveness and longevity of prevention efforts [60].

There is a need to intensify research efforts on zoonotic malaria, given the limited number (10.34%) of studies investigating knowledge of “monkey malaria”. The inclusion of *P. knowlesi* malaria in the World Malaria Report 2020 [61], necessitates a paradigm shift, urging the incorporation of zoonotic malaria into malaria program to effectively mitigate future disease burden.

For example, given the presence of natural reservoirs and the risk of contact with humans, utilizing Global Positioning Systems (GPS) for surveillance in affected areas can to identify spatial-temporal risks to the population [62]. Social science studies can facilitate in exploring underlying reasons that expose communities to the risk of zoonotic malaria [63–64]. Recognizing various challenges associated with controlling zoonotic malaria in these areas - such as social disparities, presence of reservoirs, geographical and resource constrainsts- program implementers must invest considerable time and resources to mitigate and control zoonotic malaria. Unlike human malaria control where the core vector control interventions focus on the use of ITNs and IRS, managing zoonotic malaria cases should be grounded in the One Health policy [65–66]. This policy advocates for a collaborative approach to health challenges, recognizing the interconnectedness of human, animal and environmental science. Therefore, for zoonotic malaria, programs should prioritize not only the health of humans but also the conservation of the environment and non-human primates, taking action for better health outcomes in vulnerable communities.

In addressing the dynamic environmental changes, livelihood needs of the population and the challenges in controlling monkeys and mosquitoes, the development of a vaccine becomes paramount for zoonotic malaria. The year 2021 witnessed a pivotal advancement in the fight against human malaria, specifically *P. falciparum* malaria, with the WHO recommendation of the RTS,S/AS01 vaccine for deployment in African countries with moderate to high malaria transmission [67]. Concurrently, the adoption of a vaccination strategy, complemented by enhanced surveillance and locally tailored interventions, holds considerable promise for effective zoonotic malaria control. Such a vaccine could serve as a cornerstone in the comprehensive approach needed to combat this multifaceted public health challenge. This approach is significant when considering the future threat posed by zoonotic malaria, particularly in light of escalating issues related to drug resistance against *P. falciparum* in Southeast Asia and Africa, and the growing resistance of Anopheles vectors to insecticide [68].

We identified significant methodological gaps in the literature, particularly in studies designed to investigate knowledge and local understandings of malaria. For example, a study on Palawan Island in the Philippines highlighted the advantages of using photovoice, a qualitative method of identifying people’s perceptions through photographs, providing a more accurate interpretation of their experiences and lifestyle [41]. Elsewhere, qualitative methods have explored the issues with cost, distance to health facilities, and elders’ authority as challenges to health-seeking behaviour in communities exposed to malaria in Nepal [69]. In Zanzibar, direct observation and in-depth interviews identified the gaps in malaria prevention due to routine household activities, livelihood events, and social gatherings that lasted until night time [70]. Future research should consider using qualitative methods to understand better local communities’ experiences, perspectives, and insights regarding malaria prevention.

There are numerous malaria-control challenges in the SEA and WP regions, including misconceptions about malaria, poor access to healthcare services for diagnosis and treatment, and the limited feasibility of preventive measures due to occupation, livelihood, and lifestyle. Socio-culturally sensitive strategies and community engagement in malaria planning, as well as addressing structural, political, and policy barriers that prevent access to disease prevention, need to be addressed in rural and vulnerable communities [12, 71]. Socioeconomic factors, knowledge, beliefs, and access to healthcare services and information all play a vital role in health-seeking behaviour and compliance with malaria-prevention measures. Cooperation with the Ministry of Health and other agencies is critical when addressing the threat of zoonotic malaria. There is a need for greater consensus within the malaria research community regarding knowledge, understanding, and compliance with malaria-prevention measures while offering recommendations for future research to improve the control of zoonotic malaria.

### Recommendation towards overcoming challenges to zoonotic malaria prevention

#### Role of health education and socio-behaviour changes

There is a need to address misconceptions about malaria which can potentially hinder the effectiveness of zoonotic malaria programs. Policymakers must recognize the impact of misconception on attitudes and disease-prevention practices [72]. Integrating health education into zoonotic malaria control programs is essential, enhancing disease knowledge at both individual and community levels. Communities need to be informed about the risk of zoonotic malaria exposure and perceive the susceptibility and severity of the illness.

To improve the effectiveness of current preventive measures, malaria programs should strive to understand cultural beliefs, social norms, and community practices related to malaria prevention. This understanding is crucial for implementing targeted socio-behavioural-change programs in affected communities [71]. Actively engaging communities and incorporating sociocultural beliefs into malaria prevention strategies are imperative. In remote areas with limited health facilities, traditional healers can be incorporated into malaria-control programs [73]. Innovative approaches such as drama, poetry, and singing have proven successful in promoting malaria awareness and prevention in rural areas [74]. Strengthening the capacity of community health workers is essential, particularly in remote areas vulnerable to zoonotic malaria with limited health facilities. Therefore, efficient health education is critical to improve the public’s understanding of malaria prevention and zoonotic malaria control in these regions.

#### Sociocultural considerations for zoonotic malaria prevention and control: importance of locally targeted interventions

Malaria transmission is influenced by sociocultural factors and behaviours that increase the risk of infection. Understanding these factors is crucial for effective prevention. This review emphasizes the need for culturally tailored zoonotic malaria-prevention strategies, addressing the healthcare-service inequities in vulnerable communities. Policymakers must consider local social-cultural contexts, such as social norms and daily outdoor work routines, that could hinder regular bed net usage when designing zoonotic malaria-control programs [63].

Tailored malaria-control programs are needed to target local risk factors for malaria exposure. Zoonotic malaria studies should prioritize all ages and genders, including those not working in the agricultural sector. In the Asian context, where life is centred around sociocultural influences, political situations, and the economy, research ethics must be prioritized when conducting research in communities [75]. Malaria programs across regions should consider local epidemiology, zoonotic malaria risk and socio-demographic and sociocultural characteristics. Considering gaps in zoonotic malaria control in these regions, a comprehensive study is necessary to fully understand community perspectives on different types of malaria, risk factors, and preventive measures associated with exposure and time spent outdoors.

### Community engagement and multi-sectoral collaborations

Community engagement is vital to the success of malaria control programs [71]. Healthcare workers, malaria volunteers, community leaders and chiefs can play a significant role in their community to ensure effective implementation of malaria control programs. However, the efficiency of these individuals depends on adequate training, supervision, and resources [31] ‌. Engaging local communities through community-based approaches can provide a more nuanced understanding of local perspectives and concerns related to malaria-prevention practices [76]. Community engagement can also enable the development of locally acceptable and effective zoonotic malaria intervention packages. The success of such an approach is exemplified by the tailored malaria elimination program in the Greater Mekong Subregion, characterized by high community engagement and mobilization through locally suitable strategies [55].

Enhancing community engagement in zoonotic malaria control involves community-directed approaches, empowering community members to implement programs and recruiting trained volunteers who share responsibilities [77]. The synergy of community engagement, multi-sectoral collaborations, and governmental and stakeholder political will strengthens disease control efforts [78]. Monitoring and evaluating community engagement efforts will ensure the effectiveness and sustainability of malaria control efforts [79]. Despite the importance of biomedical diagnostics and intervention technologies, community engagement remains the cornerstone of effective malaria control at the household level [55, 79]. Context-sensitive malaria control policies, addressing challenges such as language barriers and competing priorities, are crucial to reducing local malaria burdens and preventing the spread of zoonotic malaria to unaffected areas.

### Study limitations and strengths

This study focused on articles published between 2010–‍2022. These recent studies provide information about the effectiveness of malaria control programs in each country and the influence of sociocultural factors on study participants’ knowledge, and preventive practises; older articles may no longer reflect the current malaria situation, so their exclusion unlikely affected the primary findings of this review. While the initial screening identified articles published in English, backward searches identified articles published in Indonesian. This review has limited geographical coverage, so the findings cannot be generalized to other regions or populations.

Another limitation of this review is that many of the included studies were quantitative, highlighting the need for qualitative and mixed-method research to shed additional light on community perspectives. Furthermore, the increasing frequency of non-human primate malaria cases in the last ten years, in addition to earlier reports on *P. knowlesi* malaria and current reports on *P. cynomolgi* and *P. inui*, underscored the importance of being proactive in preventing exposure to the human population. This review emphasizes the limitations of current articles investigating at-risk communities regarding their awareness of ‘monkey malaria,’ perception of the disease as a health threat, and consideration of the forest as a risk area for zoonotic malaria infection.

We have also identified potential areas for future studies, such as the necessity for future research to investigate and explore the knowledge of at-risk communities regarding zoonotic malaria, their perception of the disease as a health threat, and factors such as viewing the forest and forest fringe as a risk area for zoonotic malaria transmission.

The review also highlights methodological gaps that hinder a deeper understanding of the issues that can be overcome by conducting studies using qualitative methods such as interviews or employing participatory approaches that are able to generate information by practising knowledge democracy with at risk communities [60, 76]. Given the thematic analysis approach used to generate the findings, the interpretation by the author of this review may vary from that of other readers, which opens up future discussion.

## Conclusion

This systematic review provides insights into the knowledge, local understanding, and preventive practises related to malaria among communities in WHO SEA and WP countries; however, misconceptions still exist among these communities despite a range of knowledge on malaria transmission, symptoms, diagnosis, and prevention. This review identified gaps when investigating knowledge of “monkey malaria” within these communities, emphasizing the need for further research to explore this issue. As zoonotic malaria cases rise, tailored, locally relevant interventions are crucial for at-risk areas. These findings can guide the development of effective zoonotic malaria prevention programs addressing the unique challenges and needs of vulnerable populations in WHO SEA and WP countries. The key lies in the implementation of evidence-based interventions that prioritize community engagement, respect cultural norms, and foster multi-sectoral collaborations. Through such concerted efforts, these countries can not only control the transmission of zoonotic malaria but also alleviate the burden of the disease on their populations.

### Electronic supplementary material

Below is the link to the electronic supplementary material.

Supplementary Material 1

## Data Availability

All data generated or analyzed during this study are included in this published article.

## Abbreviations

CHWs: Community health workers
VHWs: Village health workers
VMWs: Village malaria workers
IRS: Indoor residual spraying
ITNs: Insecticide-treated bed nets
KAP: Knowledge, attitude and practice
LLIHNs: Long lasting Insecticide Hammock Nets
LLINs: Long Lasting Insecticide Nets
*M.*: Macaque
MMPs: Mobile-and-migrant populations
MMWs: Mobile and migrant workers
*P*.: *Plasmodium*
RDT: Rapid diagnostic test
SEA: Southeast Asia
VMWs: Village malaria workers
WHO: World Health Organization
WP: Western Pacific
