# Supplementary material for: Knowledge, compliance, and challenges in anti-malarial products usage: a systematic review of at-risk communities for zoonotic malaria
Source: BMC Public Health. 2024 Jan 29;24:317. doi: 10.1186/s12889-024-17792-8 (PMC10823597; doi:10.1186/s12889-024-17792-8)
Supplement: Supplementary file 1 — Supplementary Material 1 [file 12889_2024_17792_MOESM1_ESM.docx]

Table 1 The eligible articles in the review

| **Ref** | **Title/ Author(s)/ Publication Year** | **Study Location** | **Study Design/ Duration** | **Study Population/ Participants** | **Study Method/ Approach** | **Findings on Knowledge** | **Findings on Preventive Practices** | **Findings on Challenges to Malaria Prevention** | **Study Quality Appraisal** |
| --- | --- | --- | --- | --- | --- | --- | --- | --- | --- |
| 1  [55] | Community engagement, social context, and coverage of mass anti-malarial administration: Comparative findings from multi-site research in the Greater Mekong sub-Region  (Pell et al., 2019) | Greater Mekong Subregion, Myanmar, Vietnam, Cambodia, and Laos PDR | Explanatory sequential mixed‑method study design  Study duration not reported | Adult villagers (*n*= 1,500) | Qualitative:  - Semi-structured in-depth interviews  - Focus group discussions (FGDs)  - Informal conversations  - Observations  Quantitative:  - Surveys | 1. Knowledge about malaria causation/‌transmission:  - 89.6% (414‍/‍462) knew malaria is transmitted by mosquitoes (*p* < 0.001).  - 12.9% (87‍/‍677) did not know the cause of malaria (*p* < 0.001).  - Asymptomatic or symptomatic malaria disease caused by a change in lifestyle and place, not via the parasite. Based on regression analysis, eating a certain food (3.5%; 18‍/‍519; *p =*0.06); rain (1.2%; 6‍/‍519; *p =*0.33); unhygienic surroundings (4.4%; 30‍/‍682; *p*= 0.55); water (9.8%; 67‍/‍682; *p*= 0.39); and forest (3.7%; 12‍/‍321; *p*= 0.73) were all insignificant.  2. Knowledge about malaria symptoms:  - Fever: 60.5% of participants reported knowing about fever (p<0.001)  -- Shivering: 65.5% of participants knew about shivering as a symptom of malaria (p=0.02)  --Vomiting: 39.8% of participants knew vomiting as a symptom of malaria (p=0.002) | - Cambodian respondents preferred wearing long-sleeved clothing and using insecticide-treated bed nets (ITNs) to prevent mosquito bites.  - Myanmar respondents described using ITNs as uncommon and negatively influenced by high nighttime temperatures that made sleeping uncomfortable when ITNs attenuated airflow.  - Cambodian respondents described antimalarial drugs, specifically those delivered as a targeted malaria elimination (TME) component, as a malaria-prevention method or individual malaria-elimination measure. | - Isolated nature of settlements  - Lack of passable roads  - Poor mobile phone coverage  - Poor infrastructure; communities access involves long and difficult journeys | High quality |
| 2  [39] | Perceptions of asymptomatic malaria infection and their implications for malaria control and elimination in Laos  (Adhikari et al., 2018 | Thappangthong and Nong districts in Savannakhet Province in Laos PDR; four villages in Nong District | Explanatory sequential mixed method study, cross-sectional design  Conducted in June and July 2015 | Adult household heads (18 years of age and older) in four study villages (*n*= 281: Oi Tan Tip = 65; Phoun Mak Mee = 74; Tha The = 82; and Xuang Tai = 60) | Quantitative: Survey  Qualitative: FGDs (*n*= 12 FGD): conducted with 100 participants from four villages and sub-villages; each FGD involved 8–‍10 participants, selected by simple randomization (i.e., lottery method) during village meetings. | 1. Knowledge about asymptomatic malaria:  - 14.2% (40‍/‍281) agreed that seemingly healthy people could have malaria parasites in their blood. 52% (146‍/‍281) disagreed, and 33.8% (95‍/‍281) were unsure.  2. Knowledge about malaria mass drug administration (MDA):  - Participants with these responses are more likely to accept the idea of asymptomatic malaria:   (i) “MDA aims to cure everyone” (adjusted odds ratio [*AOR*] = 4.6; confidence interval [CI]: 1.6–‍13.1)   (ii) “MDA is to make our community malaria-free” (*AOR*= 3.3; CI: 1.3–‍8.1)   (iii) “I will take part in future MDA” (*AOR*= 9.9; CI: 1.2–‍78.8) more likely to accept the idea of asymptomatic malaria.  3. Knowledge about malaria exposure:  - Symptomatic and asymptomatic malaria infections associated with work in the forest and living conditions.  4. Knowledge about malaria symptoms:  - FGD respondents knew malaria signs and symptoms (i.e., fever, chills, headache).  5. Knowledge about malaria:  - Elimination measures described to eliminate malaria included the use of mosquito nets, wearing long-sleeved clothing, and taking medicine when symptomatic.  - Most respondents were unaware of MDA as a malaria-elimination tool.  6. Knowledge about malaria complications:  - FGD respondents described malaria as a significant health problem. | - 97.5% (274‍/‍281) reportedly used mosquito nets at home  - 93.2% (262‍/‍281) wore long-sleeved clothing in the forest to prevent mosquito bites  - 91.1% (256‍/‍281) had slept under a mosquito net at home the previous night | - Geographical isolation and mobile population  - Living in a forested area with a high risk of mosquito-human interaction  -Suboptimal healthcare services | High quality |
| 3  [43] | Acceptability of insecticide-treated clothing for malaria prevention among migrant rubber tappers in Myanmar: A cluster-randomized non-inferiority crossover trial  (Crawshaw et al., 2017) | Sixteen clusters in Thanbyuzayat Township, Mon State, Myanmar | Explanatory sequential mixed-method study design, cross-sectional study  Cluster-randomized, double-blind, non-inferiority crossover trial to determine the accessibility of insecticide-treated clothing (ITC) versus that of non-treated clothing (NTC)  Conducted between January and May 2015 | Migrant rubber tappers  (*n*= 234 participants) | Quantitative: Survey with cluster Arm 1 (trial ITC followed by NTC), and Arm 2 (trial NTC followed by ITC)  Qualitative: FGDs (*n*= 52) with each group having 6–‍10 participants, conducted separately by gender and cluster; and IDI (*n*= 5) exploring critical topics related to clothing perceptions, preference, acceptability and use in more depth.  The intervention study used ITC and NTC.  Acceptability is evaluated through structured questionnaires, FGDs, and in-depth interviews. | 1. Knowledge about malaria:  - Participants could describe malaria or mosquito bites as malaria causes.  - Prevalent misperceptions about malaria causes, such as drinking or bathing in uncleaned or fresh water, eating certain foods, and poor hygiene and sanitation.  2. Knowledge about malaria symptoms:  - Baseline malaria awareness and knowledge were generally good as participants could correctly list symptoms.  3. Knowledge about malaria prevention:  - Participants reported high acceptability to ITC usage, which suggests ITC may be an appropriate personal-protection strategy for migrant rubber tappers in outdoor transmission settings in Myanmar. | - Most used mosquito prevention methods were long-lasting insecticide nets (LLINs) (56% across both arms) and mosquito coils (44% across both arms)  - Arm 1 household (trialled ITC then NTC) more likely to be poor (30% in lowest wealth quintile), compared to 10% in Arm 2 (trialled NTC then ITC) *p* < 0.05).  - Arm 2 respondents were likelier to use LLINs as a malaria-prevention method (*p* < 0.05).  - A higher proportion of Arm 1 respondents used wood smoke as a prevention method than Arm 2 respondents (*p* < 0.05). | -Lack of supportive financial support to sustain intervention as multiple sets of insecticide-treated clothing are required due to seasonal factor | High quality |
| 4  [47] | Knowledge, access, and utilization of bed-nets among stable and seasonal migrants in an artemisinin resistance containment area of Myanmar  (Phyo Than et al., 2017) | Four endemic malaria regions in Myanmar | Quantitative study design, cross-sectional study  Conducted in November and December of 2014 and 2015 | Seasonal and stable migrants in artemisinin-resistant containment area (*n*= 2,484)  37% were seasonal migrants | A survey using secondary data from two community-based surveys | 1. Knowledge about malaria prevention using LLINs/ITNs:  - Approximately 70% of respondents knew the importance of ITNs/‌LLINs, but less than half had specific knowledge of LLINs; ITN knowledge was significantly lower in seasonal migrants.  - More than 80% of respondents from both groups were aware of ITNs. Knowledge of bed net impregnation and ITN retreatment was poor (< 10%); knowledge of ITN retreatment was significantly lower among seasonal migrants.  2. Knowledge about bed net usage:  - Approximately half of all household members slept under bed net the previous night; this was significantly lower in seasonal migrants. | - Even though nearly all households had access to at least one-bed net per household (any type), households having at least one-bed net per two people was low: 13% of stable migrants and 9% of seasonal migrants (*p* < 0.01)  - Significant shortfall of bed nets available for household members. | 1. Access to health workers, knowledge about LLINs, knowledge of bed net impregnation and retreatment universally low.  2. Lack of reliable information on migrants’ mobility patterns in the Mekong region is also considered a hurdle to sustainable malaria-control efforts, which merits specific research. | High quality |
| 5  [44] | Perceived Role and its Enhancing Factors among the Village Health Volunteers Regarding Malaria Control in Rural Myanmar  (Aung et al., 2018) | Myanmar: Areas where the MMA project has been implemented | Quantitative study design, cross-sectional study  Study duration not reported | Village health volunteers (VHVs) who had worked at least one year (*n*= 150) were chosen using simple random sampling | Survey | 1. Knowledge about malaria control:  - 48.7% of VHVs had good knowledge of malaria control  - 72.7% had a good perception of the roles of malaria control  - This may be due to some activities that are difficult to perform, especially LLINs distribution to community and observing use thereof; some VHVs may therefore not expect to perform a good role in all tasks. | Facilitated the communities by playing their role in malaria prevention | Remote area and issues with transportation  -Lack of social support to motivate the VHVs to improve their roles in malaria prevention | High quality |
| 6  [36] | Sero-prevalence of malaria and the knowledge, attitudes and practices related to malaria prevention among indigenous people living in the central forest spine in Peninsular Malaysia: A mixed-methods study  (Kader Maideen et al., 2022) | Peninsular Malaysia | Explanatory sequential mixed-method study with a serological investigation  The study was conducted in 2020, but no duration was described | Indigenous Orang Asli adults 18 years of age and older (*n*= 284), Cases (microscopically diagnosed) and controls; they were febrile patients attending the same health facility within the same week.  Quantitative:  - Non-purposive sampling due to lack of sample frame for village population, which can fluctuate over time.  Qualitative (*n*= 13): Non-purposive sampling; participants were chosen based on their talkative nature and willingness to be interviewed in depth. | Quantitative: Survey  Qualitative: in-depth interviews  Laboratory: blood film malaria parasite (BFMP)  0% malaria prevalence in the study. Those in the middle-age group (i.e., 25–‍41 years of age) who had previously tested positive for malaria were significantly more likely to have better knowledge and attitude scores.  Significant correlations were observed between knowledge–attitude and knowledge–practice. | 1. Knowledge about malaria awareness:  - 18.3% of participants had heard about monkey malaria.  2. Knowledge about malaria causation (i.e., natural history):  - More than half of respondents were aware that those doing activities in the forest (63.4%) and involved in agricultural activities (62.0%) were at higher risk of contracting malaria.  3. Knowledge about malaria treatment:  - 95.4% sought medical treatment if unwell for more than three days.  4. Knowledge about malaria prevention:  - Participants were aware that ensuring no stagnant water (85.9%), keeping the home environment clean (81.0%), and bed net usage (79.9%) can reduce mosquitoes, thereby reducing the risk of contracting malaria. | - Majority of participants reportedly removed stagnant water (92.3%), closed windows and doors during mosquito peak biting hours (90.8%), and cut bushes around the house to reduce mosquito breeding areas (89.4%).  - Most did not use larvicides in stagnant water (82.0%) or use mosquito repellents before leaving their house (71.5%). | -insufficient understanding of their life and beliefs hinder the effort to eliminate malaria among indigenous communities | High quality |
| 7  [56] | Knowledge, attitude, and practice levels regarding malaria among people living in the malaria endemic area of Myanmar  (Aung et al., 2019) | Myanmar: Most malaria-endemic villages in Banmauk Township in Sagaing region | Quantitative study design, cross-sectional  study  Conducted in July 2018 | Household leaders (*n*= 250).  - 56.0% were 35 years of age and older  - 64.4% were male  - 88.4% earned family income up to 3 million kyats. | Structured questionnaire | 1. Knowledge about malaria:  - People 35 years of age and older likely to have poor knowledge (67.9%). Participants with annual income ≤ 3 million kyats were more likely to have poor knowledge of malaria (65.6%).  2. Knowledge about malaria prevention:  - 21.6% had good malaria practices. | - Knowledge about malaria was alarmingly low, considering new interventions in the community to strengthen malaria control practices, especially care-seeking and prevention behaviors.  - It was not revealed which preventive measures were practiced. | Despite living in areas with malaria control activities, present misconceptions and inaccurate knowledge of malaria | Medium quality |
| 8  [54] | Seroepidemiological surveillance, community perceptions and associated risk factors of malaria exposure among forest-goers in Northeastern Thailand  (Amirul et al., 2022) | Northeastern Thailand: Khamcha‑i district in Mukdahan province  Three villages:  - Ban Koh (latitude 16.5798°N; longitude 102.4791°E)  - Ban Khok (latitude 28.0024°N; longitude 202.0825°E)  - Dong Yang (latitude 15.7539°N; longitude 103.2721°E) | Explanatory sequential mixed method study design, cross-sectional study with serological study  Conducted between July and  August 2019. | Adult villagers 18 years of age and older (*n*= 345)  The median respondent age was 57 years (IQR: 47–‍64 years); more than 90% were older than 30 years of age. 51.7% of respondents were females, and gender distributions were similar across three villages (*p*= 0.212). | Quantitative: Survey  Qualitative: IDI with open-ended questions  Serological: Study | 1. Knowledge about malaria causation:  - 97.7% of respondents knew about malaria transmission via mosquito bites.  - 20.9% believed malaria could be transmitted by drinking unclean water in the forest, with the significantly highest number of Dong Yang respondents (*p* < 0.001).  2. Knowledge about malaria symptoms:  - 96% of respondents recognized fever, headache, and chills as malaria symptoms, with significant differences between study sites (*p* < 0.001).  3. Knowledge about malaria prevention:  - Ban Khok respondents showed the highest proportions of knowledge of bed-net usage to prevent malaria (100%, *p* < 0.001),  4. Malaria-treatment/‌health-seeking behavior:  - 3.8% did not use medications after contracting malaria  - 100.0% knew the importance of malaria examination after returning from the forest  - 60% sought immediate treatment at nearby clinics when malaria symptoms developed, with the highest number of Ban Koh respondents (*p* < 0.001). Contrarily, Ban Khok respondents were the highest proportion of waiting out symptoms until well (93.2%, *p* < 0.001); even though only a small proportion considered buying medication from the local shop (3.2%).  5. Knowledge about malaria complications/‌severity:  - Ban Khok respondents had the least knowledge about relapsing malaria fever (13.5%, *p* < 0.001)  6. Knowledge about drug resistance:  - Ban Khok respondents had the highest drug resistance knowledge due to incomplete adherence to antimalarial drug (97.7%, *p* < 0.001). | - 75.7% of respondents did not own bed nets as part of practice to prevent malaria spread.  Only Ban Khok respondents responded well to this practice (63.2%).  - Remarkable compliance with nightly bed net usage among those who owned them (100%).  - All respondents reported 100% indoor residual spraying (IRS) coverage by health authorities at least once during 2019.  Concerning attitudes, 73.1% of respondents lived within 500 meters of forest, with the significant majority from Ban Khok (81.2%, *p*= 0.015).  Don Yang (30.7%, *p*= 0.011) had the lowest proportion of respondents who spent the night in the forest in the past six months, while Ban Khok had the highest proportion of respondents (71.4%) who entered the forest during that time. | -Mobile population, especially those who visited forested areas where there is residual malaria transmission  -Insufficient quantity of bed nets distributed to the villages | High quality |
| 9  [29] | The use of respondent-driven sampling to assess malaria knowledge, treatment-seeking behaviors and preventive practices among mobile and migrant populations in a setting of artemisinin resistance in Western Cambodia  (Ly et al., 2017) | Cambodia: Pailin province (population: 70,482) along northern edge of Cardamom Mountains; and Veal Veang District (population: 57,523) in nearby Pursat province | Quantitative study design, cross-sectional study  Conducted between November 2010 and January 2011 | Mobile and migrant populations (MMPs) (*n*= 764 from Pailin Province and *n*= 737 from Veal Veang district)  Participants were chosen by respondent-driven sampling due to a mobile, relatively hidden population. | Survey | 1. Knowledge about malaria transmission/‌causation:  - Knowledge of malaria transmission by mosquitos was 94.4% in Pailine and 98.2% in Vael Veang  - Misinformation about means of transmission and prevention methods existed, predominantly in Veal Veang, where 24.4% mentioned dirty environment, 57.6% mentioned food or drink, and < 4% (for each of these) flies, working in sun, spirits, and contact with a sick person.  2. Knowledge about malaria prevention:  - Knowledge of malaria prevention described in various methods, including sleeping under bed nets (95.5% in Pailin and 99.1% in Veal Veang), taking preventive drugs (1.2% in Pailin and 3.6% in Veal Veang), using mosquito coils (12.7% in Pailin and 14.4% in Veal Veang), keeping house surrounding clean, covering stagnant water, closing house windows, making smoke or fire, wearing protective clothing, using mosquito repellents and sprays  - In some areas, poor hygiene, unsanitary environment (4.1% in Pailin and 24.4% in Veal Veang) and contaminated food or drink (15.2% in Pailin and 57.6% in Veal Veang) were identified as means of malaria transmission. In Veal Veang, 10% of migrant forestry workers associated malaria with forests. | Lower ITN ownership in Pailin than in Veal Veang (25.3% compared to 53.2%); higher usage at night before the survey in Pailin than in Veal Veang (57.1% compared to 31.6%). | - Barriers to accessing malaria prevention and case management common among MMPs, with marked variation by the site.  - Resolving nationwide and MMP-specific challenges will require targeted interventions considering this heterogeneity. | High quality |
| 10  [37] | Perceptions and prevention practices on malaria among the indigenous orang Asli community in Kelantan, peninsular Malaysia  (Munajat et al., 2021) | Peninsular Malaysia: Pos Kuala Betis in Kelantan State; a cluster of five villages (i.e., Angkek, Betak, Sri Galas, Lambok and Podek) in Gua Musang district in Kelantan State | Quantitative study design, cross-sectional study  Conducted between June and July 2019 | Indigenous Orang Asli people 6 years of age and older (*n*= 536) | Quantitative: Survey with closed- and open-ended questions | 1. Knowledge about malaria causation:  - 50% knew mosquitoes cause malaria. (No further description of other malaria causes provided)  2. Knowledge about malaria preventive practices:  - Nearly half knew how to prevent malaria.  3. Knowledge about malaria symptoms:  - 40% knew about fever, 14% about headaches and chills, and 10% about sweating. | - 95.2% of respondents utilized LLIN every night.  - Some did not use bed nets due to low mosquito population density and disease incidence.  - 10.4% considered an alternative treatment, including practicing traditional medicine. | -The primary economic source is from agricultural activity (e.g oil palm, rubber tapping, farming, gathering and selling forest products)  -*Anopheles* mosquitoes are present in the study area  -low perceive of threat to malaria, therefore did not use the bed nets | High quality |
| 11  [38] | Malaria prevalence, knowledge, perception, preventive and treatment behavior among the military in Champasak and Attapeu provinces, Lao P.D.R.: A mixed methods study  (Vilay et al., 2019) | Laos PDR: Two southernmost provinces, Champasak and Attapeu selected by Military Health Office | Explanatory sequential mixed-method study, cross-sectional study design  Conducted in 2017 | Military personnel (*n*= 313) | Quantitative: Survey (*n*= 313)  Qualitative: FGDs (*n*= 7) and in-depth interviews (IDIs) (*n*= 49). | 1. Knowledge about malaria causation:  - 47.4% knew *Anopheles* mosquito bite transmits malaria.  - 92% believed working in the forest put them at risk of acquiring malaria.  - 60.9% believed malaria is transmitted by drinking stream water from forests.  - 28.5% believed malaria is transmitted through coughing  - 25.5% believed flies transmit malaria.  2. Knowledge about malaria symptoms:  - 88.3%, 86.1%, 85.8%, and 85.4% knew about fever, headache, body pains, and chills are malaria symptoms.  3. Knowledge about malaria prevention:  -90.3%- believed malaria is preventable and curable but can cause illness and death.  4. Knowledge about malaria complications:  - 93.8% feared contracting malaria | - 99% used bed nets  - 91.3% used mosquito repellents  - 68.4% used wood/‌plant smoke when staying in the forest.  - 80% owned LLINs  - 10.8% used hammock nets.  - 73.6% consumed drug prophylaxis distributed in camps; drug prophylaxis was significantly associated with malaria infection (p = 0.039) compared to positive and negative cases.  Statistically insignificant associations between malaria infection and preventive measures were observed. | -Remote location of the study areas hindered adequate delivery of health messages and an inadequate supply of protection tools was provided to the communities  -Communities seek self-medication and delay health-seeking treatment due to the distance to health clinics. | High quality |
| 12  [49] | Pengetahuan, sikap, dan perilaku terhadap malaria pada masyarakat di Kecamatan Alue Bilie, Kabupaten Nagan Raya, Aceh  (Yulidar 2016) | Alue Bilie sub District, Nagan Raya District, Indonesia at  desa Ujong Lamie, desa Lamie dan desa Bate Puteh | Cross-sectional study  Conducted in 2012 | Adults of reproductive age (i.e., 17–‍45 years old of age) (*n*= 75).  - 69.3% were of reproductive age, with 13.3% male and 86.7% female. Fewer men were interviewed than women because the interview was between 10 a.m. and 2 p.m., when most men were outside the home. | Qualitative study using open-ended interview | 1. Knowledge about malaria:  - 72% knew about malaria, mosquito breeding grounds, and mosquitos bite the same blood type as transmission object. | - 100% had a good attitude about malaria by complying with and being appreciative in preventing malaria.  - 92% slept under mosquito nets; 82.7% wore protective clothing during activities outside their home; 90.7% spread larvae-eating fish in mosquito breeding grounds; and 85.3% chose to clean their home environment by burning leaves, burying cans, and closing puddles. 65.3% of respondents mentioned burning mosquito repellent. | -Presence of migrant population from North Sumatera and Jawa Island.  -Population economy depended on oil palm and rubber tree plantations.  The mosquito control products design is uncomfortable (e.g protective clothing made them feel hot) | Low quality |
| 13  [48] | Knowledge, Attitude and Practice of Relapse Malaria Patients. A Cross-sectional Study from Mandailing Natal District, Indonesia  (Tanjong et al., 2016) | Indonesia: Mandailing natal district in Sumatera | Cross-sectional study  Conducted between June and September 2017 | Of adults above 18 years old (*n*= 153), 123 persons had no relapsed malaria, while 30 had relapsed)  Inclusion criteria:  - Lived in the district for more than ten years  - 20–‍50 years of age  - Not pregnant  - Regularly visiting Primary Health Center | A survey using binary “Yes” or “No” questions (e.g., “Do you agree about indoor residual spray?”; “Do you agree to take pills regularly, use bed nets, and cover water tanks?”) | 1. Knowledge about malaria:  - 67% of participants had low malaria knowledge; no details were provided except questions about malaria transmission, type of mosquitoes and parasites, and medicine.  - 76.4%, 56.1%, and 77.3% of relapse patients had insufficient knowledge, negative attitudes, and harmful practices, respectively, while in patients with no relapsed malaria, only 30.0%, 83.3%, and 23.4% had insufficient knowledge, negative attitude and not good practices, respectively.  2. Knowledge about malaria transmission/‌causation:  - 1‑in‑3 had misconceptions about malaria transmission.  3. Knowledge about malaria symptoms:  - 1‑in‑3 had misconceptions about malaria symptoms, even though they experienced symptoms like headache, vomiting, and diarrhea.  4. Knowledge about malaria prevention:  - 66.7% of participants had poor practices to avoid malaria; no details were provided, but information related to questions on attitude and practices was evident by their agreement on malaria control programs, prevention activities and rule of taking antimalarial drug)  - 61.4% had negative attitudes about preventing malaria; no further details provided.  - Twice as many relapse patients had lower knowledge, negative attitudes and poor practices than those who did not. | There were no detailed findings on the practices. However, questions were given to participants, such as ‘Do you agree on indoor residual spray?’. | -Present misconception about malaria among the participants | Low to medium quality |
| 14  [32] | Behavioural determinants of malaria risk, prevention, and care-seeking behaviours among forest-goers in Cambodia (Phok et al., 2022) | Teuk Phos in Kampong Chhnang province and Phnom Kravanh in Pursat province, Cambodia: | Explanatory sequential mixed-method study  Conducted in August and September 2022 | Mobile and migratory forest-goers, adults 18 years of age and older of both genders  - *n*= 654 for quantitative study  - *n*= 28 for the qualitative study  Inclusion criteria:  - Those who spent at least one night in the forest in the past 30 days  - Those 18 years of age and older  - Those with febrile illness events during the past three months  - Those who provided written informed consent. | Quantitative: Survey (*n*= 675)  Qualitative: In-depth interviews (*n*= 28) | 1. Knowledge about malaria causation:  - All study participants had heard about malaria, and 98% knew mosquitoes transmit malaria.  - 14% of forest-goers thought one could become infected with malaria by drinking stream water.  2. Knowledge about malaria prevention:  - 99% perceived LLIN/‌LLIHN use as a vital malaria-prevention measure.  3. Knowledge of village malaria risk:  - Malaria is perceived as a common disease, and study participants believed most people (i.e., their family, friends, and community members, had once been infected with malaria.  4. Knowledge about malaria complications:  - 79% of forest-goers perceived malaria as harmful, and 40% believed it was difficult to cure.  - Forest-goers perceived they were at the most significant risk of malaria, recognized other villagers could become infected and malaria was dangerous to individual health and family livelihood.  - Participants knew contracting malaria could prevent forest-goers from doing their daily job and impacted their earnings and family income. | - Despite high LLIN/‌LLIHN ownership (94%), only 76% used LLIN/‌LLIHN during their last visit to the forest.  - Three preferred malaria-prevention methods were LLINs/‌LLIHNs (70%), mosquito repellents (55% spray, 46% lotion), and mosquito coils (46%).  - Low repellent use, with only 9% regularly using repellents as a preventive measure.  - Fire was a popular mosquito prevention method.  - Participants explained that forest-goers made fires even when using LLIN/‌LLIHN at night because they believed fires repel mosquitoes.  - Perceived community social norms significantly associated with LLIN/‌LLIHN usage (OR: 2.7, 96% CI 1.99–‍2.64)  - Social support from other forest-goers is also significantly associated with LLIN/‌LLIHN usage (OR: 4.9, 95% CI 1.32–‍18.12). | -The design of LLIN/LLIHN is causing discomfort among forest goers due to heat and other reasons.  -Forest goers prefer self-treat the symptoms before seeking healthcare services if the symptoms do not resolve | High quality |
| 15  [34] | Acceptability and feasibility of malaria prophylaxis for forest-goers: findings from a qualitative study in Cambodia  (Jongdeepaisal et al., 2021) | Northeastern Cambodia: Siem Pang district, which borders Laos PDR to its north and west, in Stung Treng province | Qualitative study design, cross-sectional study  Study duration in detail was not reported, except qualitative study data were collected from February 2020 to April 2021 | - Forest-goers (*n*= 27)  - Local healthcare workers, community leaders and policymakers, and trial staff in the village (*n*= 19)  - 23 trials and four non-trial participants reported visiting forested areas more than 14 days per year. Recruitment took place in villages and health centers in Siem Pang. | In-depth interviews and observations conducted in village or via online video/‌audio calls. The COVID‑19 control measures prevented face-to-face interviews. | 1. Knowledge about malaria causation/‌transmission:  - Respondents recognized the risk of malaria from forest visits  - Some described risk everywhere in the forest.  - Others specified names and described hotspots, including mountains, streams, and forest areas with certain kinds of trees (e.g., bamboo).  - Some also associated places with mosquito presence.  - Most described mosquito bites as the main cause of malaria.  - Some mentioned poor hygiene or hot weather as additional causes.  - One described past malaria infections as unavoidable with limited protection (e.g., mosquito bites unavoidable when urinating in the forest). Some respondents also felt they were at risk at their farms, describing their environment as having a lot of mosquitoes, trees, and rubbish.  2. Knowledge about malaria symptoms:  - Respondents described malaria symptoms, including headache, chills, cold, sweats, fatigue, feeling heat in their chest, and muscle pain.  3. Knowledge about malaria prevention:  - Prophylaxis with artemether-lumefantrine for forest-goers was acceptable under trial conditions.  - Three factors played a significant role:   (i) Community awareness and perception of prophylaxis effectiveness   (ii) Trust in the provider   (iii) Perceiving malaria as a local health concern  4. Knowledge about asymptomatic malaria:  - Asymptomatic malaria is generally unrecognized among forest-goers.  - Most healthcare workers and half of the trial participants knew it was possible to have malaria without symptoms. | - Forest-goers used a combination of measures, including long-sleeved clothing, sometimes with balaclava or gloves, making fire, and sleeping under mosquito nets to prevent mosquito bites in the forest.  - Most received their ITN from village malaria workers (VMWs) or health center (HC)  - Some purchased nets from the local market  - Some mentioned hammocks and blankets as alternatives when they did not bring a net into the forest.  - Due to prices and availability in Siem Pang, more used mosquito coils than mosquito repellents.  - Although no respondent described taking medicine to prevent malaria before the study, one respondent mentioned taking paracetamol from the local market to the forest in case he got sick.  - One respondent reported that in the past, villagers brought antimalarial medicines for self-treatment into the forest when there were signs of malaria without taking malaria tests. | - Forest-farming and forest-going are essential livelihood activities, thus putting people in the study area at risk of malaria  -The current vector control measures such as ITNs, coils, and repellents are inadequate to protect people from mosquitoes. | Good quality |
| 16  [35] | Opportunities and obstacles to the elimination of malaria from Peninsular Malaysia: knowledge, attitudes and practices on malaria among aboriginal and rural communities (Al‑adhroey et al., 2010) | Malaysia: Two rural areas inhabited by rural population (i.e., Padang Tengku and Benta); one forest area inhabited by aboriginal peoples (i.e., Pos Betau) | Quantitative study design, cross-sectional study  Conducted between October 2008 and May 2009 | Adults 18 years of age and older of both genders who had or had not suffered malaria crisis (*n*= 223 adults: 100 forest aboriginals and 123 from rural areas; 84 men and 139 women) | Semi-structured questionnaire | 1. Malaria causation and fewer:  - Approximately half of the aboriginal participants believed mosquito bites transmit malaria, which was significantly associated with educational level (*x^2^*= 4.244, *p*= 0.039).  - Knowledge about malaria and transmission thereof was significantly higher among rural participants than among aborigines (86.2% versus 76%, *p* < 0.01).  - 86.2% of rural participants knew about malaria.  - 70.7% believed malaria is transmitted through mosquito bites, which was influenced by education level; participants with better education demonstrated more knowledge about malaria and malaria symptoms (*x^2^*= 24.037, *p* < 0.001; *x^2^*= 4.416, *p*= 0.036 respectively).  - Malaysian participants showed more excellent knowledge about malaria transmission than Chinese and Indians (*x^2^*= 6.234, *p*= 0.013).  2. Knowledge about malaria symptoms:  - Insignificant differences observed between the two communities in terms of knowledge about malaria symptoms  3. Knowledge about preventive practices:  - Knowledge and practice of malaria-preventive measures, including insecticides and eliminating breeding areas, was significantly higher among rural populations than aborigines (*p* < 0.001).  - Aboriginal participants with past malaria infection demonstrated more knowledge of malaria symptoms than those without infection history (*x^2^*= 6.810, *p*= 0.009).  4. Malaria treatment-seeking behaviour and fewer:  - 95.1% indicated they would seek treatment from HC, which was significantly associated with participants’ educational level and age (*x^2^*= 6.236, *p*= 0.013; *x^2^*= 9.856, *p*= 0.002).  - Over one-third of sick aborigines consulted herbalists or sorcerers who provided remedies and rituals to fight evil spirits. | - Use of medicinal plants and belief in witchcraft/‌sorcery to treat febrile diseases significantly higher among the aboriginal population (*p* < 0.01).  - Rural participants' educational level, age, and race were significantly associated with practising effective preventive measures (*x^2^*= 4.634, *p*= 0.031; *x^2^*= 5.483, *p*= 0.019; *x^2^*= 7.965, *p*= 0.019). | -Inadequate knowledge of malaria among the study population, particularly aboriginals. | High quality |
| 17  [31] | Village malaria worker performance key to the elimination of artemisinin-resistant malaria: A Western Cambodia health system assessment  (Canavati et al., 2016) | Western Cambodia: Five provinces, including 36 villages and eight health centers | Exploratory sequential mixed-method design, cross-sectional study  Conducted between October 2011 and January 2012 | Qualitative study: village malaria workers (VMWs) and mobile malaria workers (MMWs) (*n*= 185), public health facility staff, village chiefs, villagers, migrants, malaria patients  Quantitative strand: (*n*= 196 VMWs and MMWs) | Qualitative:  - 60 FGDs with 416 participants. There were FGDs with VMWs/‌MMWs.  - 65 in-depth interviews conducted with community members, migrant workers, village chiefs, HC staff, VMWs, and MMWs.  Quantitative: Structured questionnaire. | 1. Knowledge about malaria causation/‌transmission:  - All participants knew malaria transmit through mosquito bites.  - Reported infection sources include poor hygiene (6.1%), dirty drinking water (4.6%), unboiled water (3.6%), and bad food (1.0%).  2. Knowledge about malaria prevention:  - Risk behaviours among VMWs/‌MMWs, such as wearing protective clothing, were significant (*p*= 0.026) in preventing malaria  - Other insignificant methods include sleeping under mosquito nets, burning leaves, using repellents and insecticide sprays.  - 20.8% deemed boiling water to be a preventive measure.  3. Knowledge about malaria treatment:  - VMWs/‌MMWs understood the crucial need for directly-observed treatment (DOT). Several challenges were raised due to transportation and difficulty relocating mobile patients.  - They also understood relapse well due to incomplete treatment regimes.  - They shared about difficulty implementing DOT among MMPs due to their high mobility and suggested village chiefs reach these individuals. | Not related to this study | -Mobile population living in a remote area | High quality- |
| 18  [46] | Awareness of malaria and treatment-seeking behaviour among persons with acute undifferentiated fever in the endemic regions of Myanmar  (Naing et al., 2017) | Malaria endemic regions in Myanmar | Quantitative study design, cross-sectional study  Conducted in 2015 | Adult households in 216 villages in 27 townships (*n*= 6,597) | Survey | Knowledge scoring: 1 (yes) or 0 (no)  1. Knowledge about malaria causation/‌transmission:  - 87.5% had heard of malaria  - 87% correctly identified mosquito bites as malaria transmission-and-prevention mode  - Poor knowledge about malaria is associated with poor treatment-seeking behaviour.  - Malaria transmission misconceptions included drinking dirty water (13%), poor hygiene (6.4%), same blood group (5.3%), sharing shelter (4%), eating bad food (3.8%), sleeping together (3%), and eating bananas (2.1%).  2. Knowledge about malaria health-seeking behaviour:  - Significant proportion sought care from informal providers (e.g 16% from family, friends, or neighbours, 16% from TV, radio or video, and 3% from teachers), and delayed seeking care for more than 24 hours.  3. Knowledge about malaria prevention:  - 90% knew malaria was preventable. Common malaria preventive methods included using mosquito nets (76%), ITNs (28.9%), and mosquito repellents (11.8%); wearing long-sleeved clothing (11.8%); drinking boiled water (5.7%); burning leaves (3%); and staying out of the forest (2.2%).  - 4.6% lacked knowledge  4. Knowledge about malaria treatment:  - 90% asserted malaria could be treated, and 85% believed modern medicine could treat the disease.  5. Knowledge about antimalarial drug resistance:  - 16% knew about antimalarial drug resistance. | - Health facility staff and VHV/‌VMW most common source of malaria information  - 22.8% of respondents with fever resorted to self-medication  - Almost 44% of respondents with fever underwent blood testing; less than half tested within 24 hours.  . | - Despite a fair amount of knowledge of malaria transmission and prevention, there was poor awareness of antimalarial drug resistance | Medium quality |
| 19  [52] | Predictors of malaria-association with rubber plantations in Thailand  (Satitvipawee, 2012) | Moo 2 village, Chaiyarat subdistrict, Thailand | Quantitative study design, cross-sectional study  Conducted between January and April 2011 | Adult households 18 years of age and older of both genders (*n*= 314; 70 were malaria-affected and 243 were malaria-unaffected) | Structured questionnaire | 1. Knowledge about malaria causation/‌transmission:  - Malaria-affected malaria villages (MVs) with misconceptions about malaria had significant malaria risks related to cause (*OR*= 2.2, 95% CI: 1.0–‍4.8, *p* < 0.05), vector (*OR*= 2.9, 95% CI: 1.5–‍5.4, *p* < 0.05) and prevention (*OR*= 2.2, 95% CI: 1.1–‍4.5, *p* < 0.05).  2. Knowledge about malaria prevention:  - ITN/‌LLIN usage substantially depends on cultural factors and defensive behaviours relevant to their occupational risk despite perceived malaria threats and perceived benefits of ITNs/‌LLINs.  - Majority of MVs had low-to-moderate scores for perceived barriers (84.6%), practising preventive measures (68.7%), malaria (64.7%), and perceived susceptibility (61%); whereas good scores were achieved for perceived benefits (91.9%), and severity (53.7%). | - Preventive practices were categorized into chemical, physical, fumigation and electrical  - Most malaria-affected MVs used physical measures (48.4% versus 42.4% unaffected), followed by fumigation (25.8% versus 38.6%)  - A lesser percentage of MVs used chemicals (17.8% versus 29.3%) and electrical (11.3% versus 21.7%) | -Present migrant population in the area, whether it is a daily routine, periodic, seasonal, or long term  -Local population travel to hilly areas for plantation | Medium quality |
| 20  [57] | Integrating child health services into malaria control services of village malaria workers in remote Cambodia: service utilization and knowledge of malaria management of caregivers  (Hasegawa et al., 2013) | Study in Kampot and Kampong Thom provinces in Cambodia | Explanatory sequential mixed method study design with a cross-sectional study  Conducted between July and September 2012 | VMWs (*n*= 36) and caregivers for children under five (*n*= 800)  An equal number of VMWs villages with malaria control services only (M) and those with malaria control and child health services (M + C) selected from each province | Quantitative: Survey with a structured questionnaire  Qualitative: Interview with 1-of-2 VMWs providing services in each study village | 1. Knowledge about malaria causation/‌transmission:  - VMWs knew mosquito bites cause malaria (98.4%)  2. Knowledge about malaria prevention:  - 99.4% knew bed nets should be used while sleeping to prevent malaria  - 7.5% understood mosquito bites should be avoided  3. Knowledge about malaria symptoms:  - VMWs knew shivering (98.9%) and high fever (97.3%) were the main malaria symptoms  4. Knowledge about malaria diagnosis:  - 99.0% knew malaria is diagnosed by blood testing  - Service quality of malaria management associated with quality of VMWs (*AOR*= 3.21, *CI*= 1.34–‍7.66) and caregiver literacy (*AOR*= 9.91, *CI*= 4.66–‍21.05)  5. Knowledge about malaria treatment:  - 85.6% knew about antimalarial drugs as a treatment method | -Not described as it is not the study's aim | -The study area is remote; therefore, communities are unable to utilize healthcare services  -Present misconception about malaria | Medium quality |
| 21  [30] | Promoting community knowledge and action for malaria control in rural Cambodia: potential contributions of Village Malaria Workers.  (Lim et al., 2012) | Kampot province Cambodia | Quantitative study design, cross-sectional study  Conducted in 2009 | Female household heads in every accessible house in the village with VMWs (*n* = 153) | Interview, administered questionnaire | 1. Knowledge about malaria cause:  - 67% had correct knowledge of malaria causes in healthcare and VMWs villages; no details about transmission or exposure were shared.  2. Knowledge about malaria symptoms:  - More than 60% correctly answered all three significant malaria symptoms in both villages; no details were shared about symptoms.  - Two villages had different levels of malaria symptom knowledge; community member knowledge of malaria symptoms was significantly lower in the VMW village than in the HC village). Over one-third could not answer all three significant malaria symptoms in HC village.  3. Knowledge about malaria prevention:  - VMWs first treated 40% of fever cases, because VMWs training focused on diagnosis and treatment with less focus on prevention and vector-control-related training, even though HC was assumed to have better disease management capacity)  - 57% of participants lived in VMW village, and 75% received treatment within one day of symptom onset, and many delayed seeking treatments until two or more days after illness onset, increasing the risk of complications.  4. Knowledge about malaria treatment:  - More than 90% of fever patients sought treatment outside their homes in both villages.  - Of those in VMV villages who sought treatment outside their home during the most recent fever episode (*n*= 104), VMWs most reported treatment source (40.4%), followed by HC (27.9%) and private clinics (20.2%).  - In HC village, HC was the most reported first treatment source (94.4%) among those seeking treatment outside their home during the most recent fever episode (*n*= 124).  - Most patients with malaria-like symptoms reported being treated with the antimalarial drug in both villages (> 80%); however, nearly half could not recall antimalarial drug name; of those who could recall antimalarial drug name, A + M was the primary drug taken in both villages, followed by Malarine.  4. Knowledge about malaria complication/‌prognosis:  - 46% in the VMW village and 54% in the HC village perceived pregnant women as vulnerable to malaria infection. | ->80% of participants with malaria-like symptoms seek for health treatment and received the antimalarial drug | -Present misconception about malaria in the communities  -remote area causing difficult accessibility to the health clinic | Medium quality |
| 22  [33] | Malaria knowledge, preventive actions, and treatment-seeking behaviour among ethnic minorities in Ratanakiri Province, Cambodia: A community-based cross-sectional survey  (Yasuoka et al., 2018) | Cambodia: 62 rural villages in Ratanakiri province | The concurrent mixed-method study, cross-sectional study  Conducted in December 2015 | Mothers in 10 ethnic minority groups and Khmer group with children under two years of age (*n*= 377) | 1. Qualitative:  - Face-to-face interviews  2. Qualitative:  - Semi-structured questionnaire | 1. Knowledge about malaria causation/‌transmission:  - 40.6% knew the precise malaria transmission route  - 29.2% knew about mosquito breeding places  - Staying overnight at a farm hut was significantly associated with fever during the most recent pregnancy (*AOR* = 2.008, 95% CI: 1.215–‍3.321) and with a greater likelihood of the child experiencing fever (AOR 3.681, 95% CI 1.943–‍6.972).  2. Knowledge about malaria symptoms:  - 44.6% were aware of malaria symptoms.  3. Knowledge about malaria-treatment-seeking behaviour:  - 26.3% of mothers had fever during their most recent pregnancy.  - 37.4% of HC and 12.1% of VMWs sought treatment from public providers  - Approximately 40% of mothers did not seek treatment.  - Of those who sought treatment (*n*= 60), more than half (57.9%) sought treatment the same day or the day following fever onset.  4. Knowledge about malaria diagnosis:  - 60% were confident they received blood tests for malaria diagnosis, and five were positive for malaria (three *P. falciparum c*ases and two *P. vivax* cases). | - Most mothers slept under bed nets at home (95.8%) and wore long-sleeved clothing (83.8%) to prevent malaria  - Mothers participating in various malaria-preventive actions protected against fever in children (AOR 0.292, 95% CI: 0.136–‍0.650).  - Among those experiencing fever during pregnancy, 39.4% did not seek treatment. | This study highlights the need to spread accurate malaria knowledge, raise awareness of health risks related to agricultural practices and promote treatment-seeking behaviour among ethnic minorities to strengthen malaria-elimination engagement. | High Quality |
| 23  [40] | Determining the impact of community awareness-raising activities on the prevention of malaria transmission in Palawan, the Philippines  (Matsumoto–Takahashi et al., 2014) | Palawan Island: 20 remote malaria-endemic villages throughout Palawan located in four high malaria-endemic provinces | Quantitative study design, cross-sectional study  Conducted from January to February 2012 | Adult patients with a history of malaria (*n*= 141). | Structured interviews ( | 1. Knowledge about malaria transmission:  - Study did not provide detailed malaria transmission knowledge but identified significant differences between northern, central, and southern regions (*p* < 0.001).  - Knowledge about vector species and most active times of vectors was insignificant (*p*= 0.142 and *p*= 0.371, respectively).  2. Knowledge about malaria symptoms:  - Knowledge of malaria symptoms was insignificant between all three regions (*p*= 0.192)  3. Malaria prevention knowledge from participating in awareness-raising malaria-prevention activities:  - Tagalog ethnicity is positively associated with better self-implemented preventive measures against malaria  - 100% reported sleeping under bed nets at home  - 60.9% returned home before dawn  - 60.9% wore long-sleeved shirts and pants  - 30.1% refrained from going to the forest (51.9% brought hammock nets, and only 15.0% always brought them into the forest).  - Participants who participated in awareness-raising malaria-prevention activities with microscopists were found to enact more preventive measures than those who did not receive such activities.  - Participants who expressed higher satisfaction with their microscopists also took more preventive measures against malaria than those who reported dissatisfaction with their microscopists. | While all participants reported always sleeping under bed nets at home, 30.1% needed to go or stay in the forest to tend gardens, and only 15.0% always brought mosquito nets into the forest. | -The community resides in a remote and hilly area | Medium quality |
| 24  [42] | Low perception of malaria risk among the Ra‑glai ethnic minority in south-central Vietnam: Implications for forest malaria control  (Peeters Grietens et al., 2010) | Hilly and forested areas in the south-central region of Vietnam | Multimethod study design, cross-sectional study  Conducted between July 2005 and September 2006 | Ra‑glai ethnic minority group, with no age or gender description  Subsequently | Qualitative: Ethnography (*n*= 101 households; 58 informants were selected during participant observation at public spaces), and 43 were selected due to a recent malaria episode in one household member (detected by PCD).  Quantitative: Malariometric survey, (*n*= 3,685) randomly selected individuals | 1. Knowledge about malaria:  - Half of the population could identify illness by name in Vietnamese or Ra‑glai.  - 75% were unsure of the cause of their most recent fever.  - Diagnosed malaria patients largely unaware of specific fever they had recently suffered.  2. Knowledge about malaria causation/‌transmission:  - 15.6% acknowledged a higher risk of contracting malaria in the forest than in the village  - respondents were aware of the risk of mosquito bites in the evening and early morning, corresponding to when insects were more visible and a more significant nuisance. However, they typically believed few-to-no mosquitoes were present for the remainder of the night.  3. Knowledge about malaria prevention:  - 84.6% regularly used bed nets in villages  - 52.9% slept under bed nets in forest fields.  - 41.6% regularly used bed nets in village and forest fields.  - 20.6% slept unprotected in both places. | Bed nets usage was irregular in villages and forest fields. Even when bed nets were used, not all household members slept under one. | - Main constraints to consistent bed net use were:   (i) Bed nets cast aside when nighttime temperatures are too hot.   (ii) Bed nets increased distance to fire in cold temperatures and humid weather.   (iii) Bed nets could catch fire while sleeping.   (iv) Poorer families lacking the means to buy blankets and covered themselves with bed nets.   (v) Bed nets are often damaged in impoverished living conditions, and people cannot afford to purchase extra nets.  An effective malaria intervention should consider the socio-cultural aspects of the community, including improving poverty-related barriers rather than relying solely on public health messages, strengthening the health system and structural factors | Good quality |
| 25  [41] | Re-imaging malaria in the Philippines: how photovoice can help to re-imagine malaria  (Iskandar, 2017) | Bataraza municipality in the Philippines was highly endemic (≥ one case per 1,000 population) | Quasi-experimental design: Control and intervention group  No description of year/months however, information described for each study:  (1) Photovoice study: 15-week  (2) Post photovoice (intervention) questionnaire was done 3 months afterwards | School children from four elementary schools in two *barangays*, Inogbong and Bonobono | Photoice study: 15-week with 108 school children from 44 predominant ethnicities in Palawan school.  - 83 adults/‌caregivers from every child’s household. | 1. Knowledge of malaria causation/‌transmission and its sources (e.g., mosquitoes):  - Misconceptions about malaria aligned with local beliefs about body, health, and well-being; health practitioners often overlooked this.  2. Knowledge of malaria symptoms (e.g., fever)  3. Knowledge of preventive practices (e.g., mosquito nets).  4. Knowledge of diagnostic practices (e.g., use of microscopy and rapid diagnostic tests)  5. Knowledge of treatment practices (e.g., use of antimalarial drugs). | The study described malaria prevention through various approaches  (i) Avoiding mosquitoes landing on your hand  (ii) cleaning the surrounding area, including throwing the dirty water and avoiding rubbish  (iii) make smoke regularly to avoid mosquitoes  (iv) burning the garbages to avoid mosquitoes breeding areas | -  Funding supporting malaria programs should be decentralized and delivered locally through rural health clinics and stations. | High quality |
| 26  [59] | A comparison of malaria prevention behaviors, care-seeking practices and barriers between malaria at-risk worksite migrant workers and villagers in Northern Shan State, Myanmar—a mixed method study  (Aung, 2022) | Four targeted townships and villages (*n*= 20) located nearest to worksites (*n*= 23) Malaria endemic area in eastern Myanmar near China border | Explanatory sequential mixed method study design, cross-sectional study  Conducted in March 2019 | - Migrant workers  - Villagers  - Key stakeholders | Quantitative: migrant workers (*n*= 880)and villagers (*n*= 447)  Qualitative: In-depth interviews (*n*= 14) with key stakeholders, including worksite managers and village leaders. | 1. Knowledge about malaria prevention:  - Sleeping under bed nets are common in worksites (73.9%) and villages (85%).  - ITN/‌LLIN usage is lower in worksites (39%) than in villages (80%).  - 30.4% of worksites and 65% of villages burned incense or coils to mitigate mosquito bites.  - 8.7% of worksites and 30% of villagers burned leaves to eliminate mosquito reservoirs and repel mosquitoes.  - 4.35% of worksite workers used insect repellents, and 15% of villagers applied them to mitigate mosquito bites.  - 47.8% of worksites and 75% of villages had residents wearing protective clothing to prevent mosquito bites.  - Malaria prophylactic drug usage is no longer popular; 4.35% of worksites and 10% of villages had adopted this practice.  - Villagers commonly used local belief-driven traditional practices.  2. Knowledge about malaria health-seeking treatment:  - Self-medication is popular among worksite workers and villagers due to easy access to Western medicine.  - Villagers and workers sought care at government hospitals only for severe conditions.  - Eight determinants influenced the health-seeking behaviour of worksite workers and villagers when experiencing fever:   (i) Local and traditional beliefs   (ii) Easy availability   (iii) Arrangement by worksites   (iv) Local recommendation   (v) Service cost   (vi) Accessibility   (vii) Trust and relationship   (viii) Disease severity  3. Knowledge about malaria treatment:  - Traditional antipyretics used. | - Treatment by traditional means is both respondents' most reported initial treatment option.  - Treatment by traditional means included taking traditional or local medicine and herbs by themselves or recommended by coworkers or villagers and doing traditional practices called *Makalaung*, the act of piercing fingertips and toe tips with a needle, or *Sar‑chit*, the act of scratching with a sharp object until skin is red.  - They believed these activities could relieve fever.  - Some sought treatment from shamans due to spiritual and traditional beliefs.  - Traditional methods are common among villagers due to local beliefs. | -Remote communities  -Socio economic barriers are causing issues with health-seeking treatment. There is insufficient income and no health coverage for workers  -In affordability to pay for transportation costs to seek medical care at health centres, including paying for the healthcare services  -Daily wage earners hinder their motivation to seek healthcare when they are sick  -Negative experiences with healthcare workers and fear of visiting healthcare centres  -Inadequate screening (as only rapid test was provided), and not another diagnostic method at the healthcare centres | Medium quality |
| 27  [51] | Knowledge, attitudes, and practices of *Anopheles* mosquito control through insecticide treated nets and community based health programs to prevent malaria in East Sumba Island, Indonesia  (Bandzuh et al., 2022) | Indonesia: East Sumba Regency | Qualitative study design, cross-sectional study  Conducted between November 11 and November 19, 2015 | All FGD participants were 18 years of age and older, primarily women. Only one FGD comprised of men (i.e., 7 men in Kota Waingapu).  Key informant interviews (*n*= 14) were conducted with religious leaders, health workers, and women group leaders. | Qualitative:  1. FGDs (*n*= 7) stratified by urban or rural location and level of malaria transmission. Each group varied from four participants in Kota Waingapu to eight participants in Umalulu. Each group had different occupational backgrounds (e.g., homemakers, community leaders, employed outside their homes, and government officers)  2. IDIs (*n*= 14)  All interviews employed a semi-structured protocol | 1. Knowledge about malaria prevention:  - ITNs are perceived to prevent malaria effectively and are considered acceptable.  - Some did not always use ITNs.  - Some participants perceived insecticides as ineffective because they lived in open houses on stilts, and insecticide dispersed everywhere when spraying, so they preferred mosquito coils.  2. Knowledge about women’s role:  - Integrated health service posts (*posyandu*), and community-based health workers (*kaders*) in combating malaria  - Participants discussed women’s important role in information dissemination related to mosquitoes and mosquito-borne diseases and distribution and use | - Participants shared about traditional malaria preventive methods:   (i) Putting papaya leaves under the bed   (ii) Burning breadfruit leaves or fruit   (iii) Consuming papaya, neem, and betel leaves by boiling them and drinking the water  - Some participants discussed using commercial oils, including Nona Mas, Autan, Soffell and telon oils. | Many challenges shared related to ITN usage, including environmental conditions, such as high temperature, smell and feeling claustrophobic. | High quality |
| 28  [50] | Defining malaria risks among forest workers in Aceh, Indonesia: A formative assessment  (Ekawati et al., 2020) | Aceh province of Indonesia from June to August 2016 | Qualitative study design, cross-sectional study | Forest workers  Key informants consisted of nine groups; index case defined as those diagnosed with malaria at primary healthcare between 2014–‍2015, resided in 1‑of‑4 subdistricts, and 18 years of age and older; and their coworkers (i.e., 18 years of age and older), community health workers, community members who lived in forest fringe and non-forest fringe, forest-worker employers, health staff working in a health facility, and community leaders | 1. FGDs (*n*= 5)  2. In-depth interviews (*n*= 18) | 1. Knowledge about malaria exposure/‌transmission:  - Participants shared work activities, including mining, logging, and agriculture in deep forests and along forest fringe.  - Forest workers, mainly miners and loggers, often spent weeks and months at worksites in makeshift housing.  - Some participants believed malaria risk was limited to those who work or stay overnight in forests or mountains; thus, those living in villages faced a lower risk of malaria.  - Sleeping outside and spending time in cold places in simple huts without mosquito nets is mentioned as a reason for contracting malaria.  - Participants described greater malaria risk for men than children.  - Participants described monkey sightings, specifically long-tailed macaques, southern pig-tailed macaques, and Sumatran *surili*, at all study sites.  2. Knowledge about malaria symptoms:  - All participants could describe malaria symptoms, including headache (*n*= 39); chills (*n*= 33); fever (*n*= 31); muscle and joint pain (*n*= 30); feeling hot and cold (*n*= 29); weak (*n*= 21); vomiting (*n*= 11); nausea (*n*= 8); and dizziness (*n*= 7).  3. Knowledge about malaria treatment and diagnosis:  - Self-medication and seeking care from traditional healers or pharmacies, rather than health facilities.  - Most forest work locations lived within one-day journey of health clinics.  4. Knowledge about malaria prevention:  - Participants reported low preventive-practice rates among forest workers, including using bed nets, mosquito coils, repellents, and medication.  - Some participants shared traditional practices, such as consuming papaya leaves, bitter melon, mahogany seeds, cat whiskers, and lanzone root.  - Other traditional practices included use of traditional herbs (*jamu*) and wearing charmed stones to protect against mosquito bites. | - Participants stated forest workers rarely utilized mosquito prevention measures due to the work environment, and bed nets were often out of stock and expensive.  - Participants also irregularly used bed nets due to breathing difficulty and feeling hot and uncomfortable.  - Some believed chemicals used to treat bed nets resulted in odours irritating eyes and skin.  - Some forest workers viewed prevention as unnecessary because they could buy medication whenever they felt ill. | Some forest workers viewed prevention as unnecessary because they could buy medication whenever they felt ill. | High quality |
| 29  [45] | The Determinants of Delayed Diagnosis and Treatment Among Malaria Patients in Myanmar: A Cross-Sectional Study  (Than et al., 2019) | - Paletwa township  - Chin State in upper Myanmar  - Ann township (i.e., Rakhine State) in middle Myanmar  - Bokpyin township, Tanintharyi division in lower Myanmar | Quantitative study design, cross-sectional study  The study duration was not described | Working-age adults diagnosed with malaria in the past year (*n*= 220) | Survey | 1. Knowledge about malaria:  - 80% had poor knowledge of malaria, and 20% had good knowledge.  2. Knowledge about malaria diagnosis and treatment:  - More than half of the participants visited HCs or volunteered for the first treatment of choice.  - Significant associations were observed between age, gender, education, marital status, occupation, and delayed diagnosis and treatment (*p* < 0.05)  - Approximately 39.5% delayed diagnosis, and 42.8% delayed treatment more than 72 hours after symptom onset.  - Higher proportion of respondents with low knowledge of malaria (48.9%) delayed diagnosis 72 hours longer than those with high knowledge (2.3%).  - Higher proportion of respondents with low knowledge of malaria (52.8%) delayed treatment 72 hours longer than those with high knowledge (2.3%).  3. Knowledge about social support:  - A higher proportion of respondents who obtained low-to-moderate social support for malaria services (49.4%) delayed diagnosis 72 hours or longer than those who did not (4.2%). | Not described as it was not the study's aim | -Poor knowledge of malaria among the participants, therefore, delaying health seeking  Working in remote areas and having difficulty seeking healthcare treatment  -No nearby health facilities that could offer diagnosis and treatment  -Unaffordability to pay for transport to seek healthcare | Low-to-medium quality |
